# Biomarkers for tissue engineering of the tendon-bone interface

**DOI:** 10.1371/journal.pone.0189668

**Published:** 2018-01-03

**Authors:** Lara A. Kuntz, Leone Rossetti, Elena Kunold, Andreas Schmitt, Ruediger von Eisenhart-Rothe, Andreas R. Bausch, Rainer H. Burgkart

**Affiliations:** 1 Klinik für Orthopädie und Sportorthopädie, Klinikum rechts der Isar, Technische Universität München, München, Germany; 2 Lehrstuhl für Zellbiophysik, Technische Universität München, Garching, Germany; 3 Center for Integrated Protein Science (CIPSM), Department of Chemistry, Technische Universität München, Garching, Germany; Ecole normale superieure de Lyon, FRANCE

## Abstract

The tendon-bone interface (enthesis) is a highly sophisticated biomaterial junction that allows stress transfer between mechanically dissimilar materials. The enthesis encounters very high mechanical demands and the regenerative capacity is very low resulting in high rupture recurrence rates after surgery. Tissue engineering offers the potential to recover the functional integrity of entheses. However, recent enthesis tissue engineering approaches have been limited by the lack of knowledge about the cells present at this interface. Here we investigated the cellular differentiation of enthesis cells and compared the cellular pattern of enthesis cells to tendon and cartilage cells in a next generation sequencing transcriptome study. We integrated the transcriptome data with proteome data of a previous study to identify biomarkers of enthesis cell differentiation. Transcriptomics detected 34468 transcripts in total in enthesis, tendon, and cartilage. Transcriptome comparisons revealed 3980 differentially regulated candidates for enthesis and tendon, 395 for enthesis and cartilage, and 946 for cartilage and tendon. An asymmetric distribution of enriched genes was observed in enthesis and cartilage transcriptome comparison suggesting that enthesis cells are more chondrocyte-like than tenocyte-like. Integrative analysis of transcriptome and proteome data identified ten enthesis biomarkers and six tendon biomarkers. The observed gene expression characteristics and differentiation markers shed light into the nature of the cells present at the enthesis. The presented markers will foster enthesis tissue engineering approaches by setting a bench-mark for differentiation of seeded cells towards a physiologically relevant phenotype.

## Introduction

Interfaces between mechanically dissimilar materials are prone to stress concentrations and the failure risk is increased. Entheses are hard-soft interfaces between tendon/ligament and bone and are subject to extraordinary high mechanical demands. Surgical reconstruction of entheses is very challenging and the regenerative capacity of these biological hard-soft interfaces is low. Wound healing at the interface region mostly results in tissue with lower structural integrity and mechanical functionality than before injury[1]. Furthermore, the incidence of re-tears of surgically fixed entheses is very high[2, 3]. Approaches to successfully regenerate the functional integrity of entheses are clinically crucially needed.

There has been an increasing interest in enthesis tissue engineering as strategy to overcome the limits regarding regeneration of tendon/ligament-bone interfaces. Enthesis tissue engineering is challenging due to structural and compositional complexity of the enthesis[4] as well as limited understanding of developmental and cellular processes at the interface[5]. Several approaches to enthesis tissue engineering have been reported that range from artificial biomaterial scaffolds[6–9] to decellularized donor graft scaffolds[10, 11]. Most approaches to enthesis tissue engineering include a scaffold in combination with cells that are seeded onto the scaffold. Due to their potential to differentiate towards multiple cell lineages, mesenchymal stem cells have been regarded as very promising for enthesis tissue engineering. However, the molecular pathways and differentiation markers that characterize physiological relevant enthesis cells have not been elucidated. Biomarkers are urgently needed that allow to evaluate whether scaffold seeded mesenchymal stem cells differentiate towards the physiological phenotype of interface cells to ensure functional integrity.

Transcriptomics using next generation sequencing offers the potential of precisely characterizing gene expressional levels and differentiation markers of cells. Here, we present a transcriptomic study that characterized cellular features at the tendon-bone interface as well as detected biomarkers for tendon-bone interface tissue engineering. The expression patterns of cells residing within the tendon-bone interface were analyzed and these expression patterns were compared to expression patterns of cells residing within Achilles tendon and cartilage of the tibial plateau to identify differentiation markers for the respective cells.

The presented transcriptome study expanded our previously published proteome study[4] and allowed to discover strong interface cell biomarkers by combining transcriptomics with proteomics data. The presented integrative study provides insights into the differential gene expression properties of cells present at the tendon-bone interface. The identified biomarkers allow for a better understanding of the cellular properties of the enthesis and offer great potential for use in enthesis tissue engineering.

## Materials and methods

### Confocal microscopy

Achilles tendon-bone insertion (enthesis) samples were dissected from porcine legs obtained from a local abattoir. Samples were frozen at - 20°C in Dulbecco’s Modified Eagle Medium (Sigma-Aldrich) and cut into sagittal 2 mm slices using a diamond coated band saw (Exakt 300CL). Enthesis slices were fixed in 4% paraformaldehyde for 48 h and subsequently washed with water. Samples were decalcified using a custom-made decalcification solution (0.27 M citric acid / 0.1 M EDTA / PBS) for 4–6 weeks under continuous agitation. Decalcified enthesis slices were cryocut sectioned into 7 μm thick sections using Cryo-Star HM 560V (ThermoScientific), air-dried, and stored at - 80°C. For staining, cryocut sections were thawed and air dried for 15 min. Sections were fixed in a mixture of 1:1 (vol/vol) acetone:methanol for 15 min and then air dried for 15 min. Samples were rehydrated with phosphate buffered saline (PBS, pH 7.4) for 10 min. Rehydrated samples were stained for cells using SYTO^®^ 13 (ThermoFisher Scientific) or Yo-Pro^®^-1 (ThermoFisher Scientific). SYTO^®^ 13 or Yo-Pro^®^-1 were 1:1000 diluted in PBS and 70 μl dilution was applied per cryocut section. Samples were incubated for 20 min at RT in the dark. Subsequently, samples were washed with PBS-Tween^®^ 3 x 5 min, washed in PBS for 5 min, and covered using Fluorescence Mounting Medium (Dako) and high precision microscope cover glasses (Marienfeld). Imaging was performed using an inverted laser-scanning confocal microscope (Leica TCS SP5) with a 63 x oil objective, NA = 1:4 (Leica HCX PL APO). Sample areas with up to the order of 80 mm^2^ were imaged by acquiring tiles of sub-images. Stacks of images were acquired with 5–10 slices in z covering a thickness of 7–20 μm. Stacks were z-projected and the sub-images stiched by the Leica software. For all images the fluorescence signals of the fluorophores were detected as well as the reflection signal from the excitation laser. Images were analyzed using ImageJ[12].

### Transcriptomic analysis

The transcriptome study was performed to identify biomarkers of enthesis cells for tissue engineering approaches. The transcriptome of cells present at the enthesis was characterized and compared to the transcriptome of tendon cells. Since a previous proteome study identified cartilage markers within the extracellular matrix of the enthesis, the enthesis transcriptome was further compared to the transcriptome of cartilage cells.

### RNA extraction

Legs of six months old pigs (N = 9) obtained from a local abattoir were dissected within 5 hours after slaughter. Skin tissue, fat tissue, and muscle tissue were carefully removed using a scalpel. Achilles tendon as well as enthesis were freed by removing adjacent tendon tissue. enthesis, Achilles tendon, and cartilage samples were carefully excised using scalpels. Fat tissue and paratenon were carefully removed before retrieving enthesis and Achilles tendon samples. Enthesis tissue was completely excised along the entire interface region between Achilles tendon and bone, and pooled per sample to reduce differences that may occur due to extraction from medial or lateral location. Achilles tendon tissue was excised ~ 1.5 cm cranial of the enthesis and also extracted along the whole cross-section of the Achilles tendon, to reduce differences that may occur due to extraction from medial or lateral location. At the enthesis, Achilles tendon tissue was carefully removed as much as possible prior to taking enthesis biopsies. Since the enthesis is not fully distinguishable from the tendon without prior staining and tissue has to be fresh and unstained for RNA extraction, tendon residuals possibly remain in the enthesis samples. Cartilage tissue was excised from cartilage of the porcine tibial plateau and also pooled per sample to reduce differences that may occur from medial or lateral location. Tissue samples were transferred into cryotubes, covered with PBS, and shock frozen in liquid nitrogen. Shock-frozen tissue samples were transferred into - 80°C cold pre-cooled RNA*later*®-ICE Frozen Tissue Transition Solution (ThermoFisher Scientific) to stabilize the RNA within the tissue. Samples with RNA*later*®-ICE Frozen Tissue Transition Solution were incubated at - 20°C for at least 16 h to ensure proper diffusion of the RNA stabilizing solution into the tissue. Afterwards, stabilized samples were entirely cut into 7 μm thick cryocut sections using a cryostat HM 560 (Thermo Scientific Microtom) at -21°C. Cryocut sections were then stored in RNA*later*®-ICE Frozen Tissue Transition Solution until use. For RNA extraction, sample cryocut sections were taken out of the stabilizing solution and residual solution was quickly removed from the cryocut sections by dipping the sample onto a paper towel. For each extraction using a Qiagen RNeasy Fibrous Tissue Mini Kit, ~ 25 mg of cryocut sections per sample were suspended in 300 μl Buffer RLT containing β-mercaptoethanol according to the manufacturer’s instructions. Samples with RLT buffer were transferred to 2 ml Eppendorf tubes containing one autoclaved stainless steal bead each (bead diameter 5 mm). Homogenization was performed with a TissueLyser LT at cycles of 2 x 2 min at 20 Hz, followed by 2 min at 30 Hz and subsequently 1 min at 40 Hz. If tissue was not sufficiently homogenized, samples underwent another homogenization cycle for 1 min at 40 Hz. Lysates were transferred into new microcentrifuge tubes and 590 μl RNase-free H_2_0 as well as 10 μl proteinase K solution were added and thoroughly mixed. Proteinase K digestion was incubated for 10 min at 55°C. On-column DNase treatment was performed at 30°C for 15 min and RNA extraction was performed according to manufacturer’s instructions of the Qiagen RNeasy Fibrous Tissue Mini Kit. RNA was eluted twice in the same volume of 30 μl RNase-free water and immediately frozen at -80°C.

### RNA quality check

Integrity of extracted RNA was assessed during RNA extraction protocol development and for final RNA quality check prior to next generation sequencing. RNA integrity number (RIN) that quantifies RNA integrity was determined using an Agilent 2100 Bioanalyzer system (Agilent Technologies) and the RNA 6000 Nano Assay. Following the instructions of the Agilent RNA 6000 Nano kit, bioanalyzer electrodes were decontaminated before each measurement using RNase ZAP^TM^ (Sigma-Aldrich). Agilent RNA nano Labchips® were primed with gel-dye mix that was produced following the protocol of the Agilent RNA 6000 Nano kit. RNA samples and RNA ladder were heat-denatured for 2 min at 70°C to minimize secondary structures. The chips were loaded and subsequently vortexed at 2200 rpm using a vortex mixer MS3 (IKA). Loaded chips were transferred to the 2100 Bioanalyzer system and “Eukaryote Total RNA Nano Series” RNA assay was performed in combination with 2100 Expert Software (Agilent). RNA with RIN ≥ 7 passed quality check for next generation sequencing. RNA extraction was performed with samples from nine pigs including 2–3 technical replicates with regard to RNA extraction. 3 pools each for enthesis samples and tendon samples and 2 pools for cartilage samples were produced by mixing RNA that passed quality check. Each pool consisted of RNA from three biological replicates and from 2–3 extractions per biological replicate. Overall, each of the 8 pools contained RNA from 5–9 different RNA extractions.

### Next generation sequencing

Random primed cDNA libraries were produced and analyzed by GATC Biotech (ISO 17025 accredited). Poly(A)^+^ RNA was isolated from total RNA samples and was fragmented. Fragmented mRNA was transcribed into random-primed cDNA library. cDNA synthesis was performed with random hexamer priming. Adaptors were ligated to cDNA strands and cDNA was amplified via polymerase chain reaction.

Single end cDNA sequencing was performed on a Genome Sequencer Illumina HiSeq next generation sequencing system in sequence mode HSHOv4 SR50. A total of 30 million single reads were performed with a read length of 1 x 50 base pairs.

### Bioinformatics

The sequencing reads were aligned to the pig (Sus scrofa) reference genome Sscrofa10.2 (http://www.ensembl.org/Sus_scrofa/Info/Index) with annotations Sscrofa10.2.86 using Bowtie[13] generating genome alignments. TopHat identified potential exon-exon splice junctions of the initial alignment. Subsequently, Cufflinks identified and quantified the transcripts from the preprocessed RNA sequence alignment assembly. Cuffmerge merged the identified transcript fragments to full length transcripts. Then, full length transcripts were annotated based on the given genome annotations Sscrofa10.2.86. To determine differential mRNA expression levels, merged transcripts from enthesis, cartilage, and tendon samples were compared using Cuffdiff. Alternative splice variants as well as single nucleotide polymorphisms and insertion/deletion mutations were assessed based on existing gene models for eukaryotes.

### Functional classification

Identified genes and proteins were classified using PANTHER Gene ontology, DAVID bioinformatics resources 6.8., and networks were predicted with STRING database.

### Functional classification using PANTHER gene ontology

Gene ontology (GO) analysis was performed using PANTHER Classification System[14, 15]. PANTHER reactome pathways overrepresentation test (released 2016-07-15) with reactome version 58 (released 2016-12-07), Sus scrofa as reference list, and Bonferroni correction was used to categorize genes detected in the tissues for overrepresentation of reactome pathways. Overrepresentation of biological processes was investigated using PANTHER overrepresentation test (released 2016-07-15), PANTHER version 11.1 (released 2016-10-24) for slim biological process and GO ontology database (released 2016-12-28/2017-01-26) for biological process complete, Sus scrofa as reference list, and Bonferroni correction.

### Functional classification with DAVID bioinformatics resources 6.8

To identify candidates with transcription factor activity or growth factor activity, the Functional Annotation Tool of DAVID bioinformatics resources 6.8 (NIAID/NIH) was used[16]. Lists of genes at least twofold enriched in one of the tissues were uploaded to screen ensemble gene IDs, official gene names and entrez gene IDs. Sus scrofa was used as background. For cartilage candidates with official gene names, Homo sapiens was used as background due to lack of annotation for Sus scrofa. Functional annotation tables were exported for GOTERM_MF_FAT. Functional annotation tables were manually screened to identify candidates with associated GO terms involving transcription factor activity or growth factor activity.

### Network prediction using STRING

Protein-protein interactions between growth factors and transcription factors identified with the Functional Annotation Tool of DAVID bioinformatics resources 6.8 were predicted using STRING Database[17, 18] version 10.0. Lists of proteins were entered via “multiple proteins” function and investigated for protein-protein interaction using Sus scrofa as reference. Protein-protein interaction networks were characterized using active interaction sources textmining, experiments, databases, co-expression, neighborhood, gene fusion, and co-occurrence. Default setting medium confidence (0.400) was defined as minimum required interaction score. Networks were exported with confidence view settings in which line thickness indicates strength of data support.

## Results

**Fig 1:**
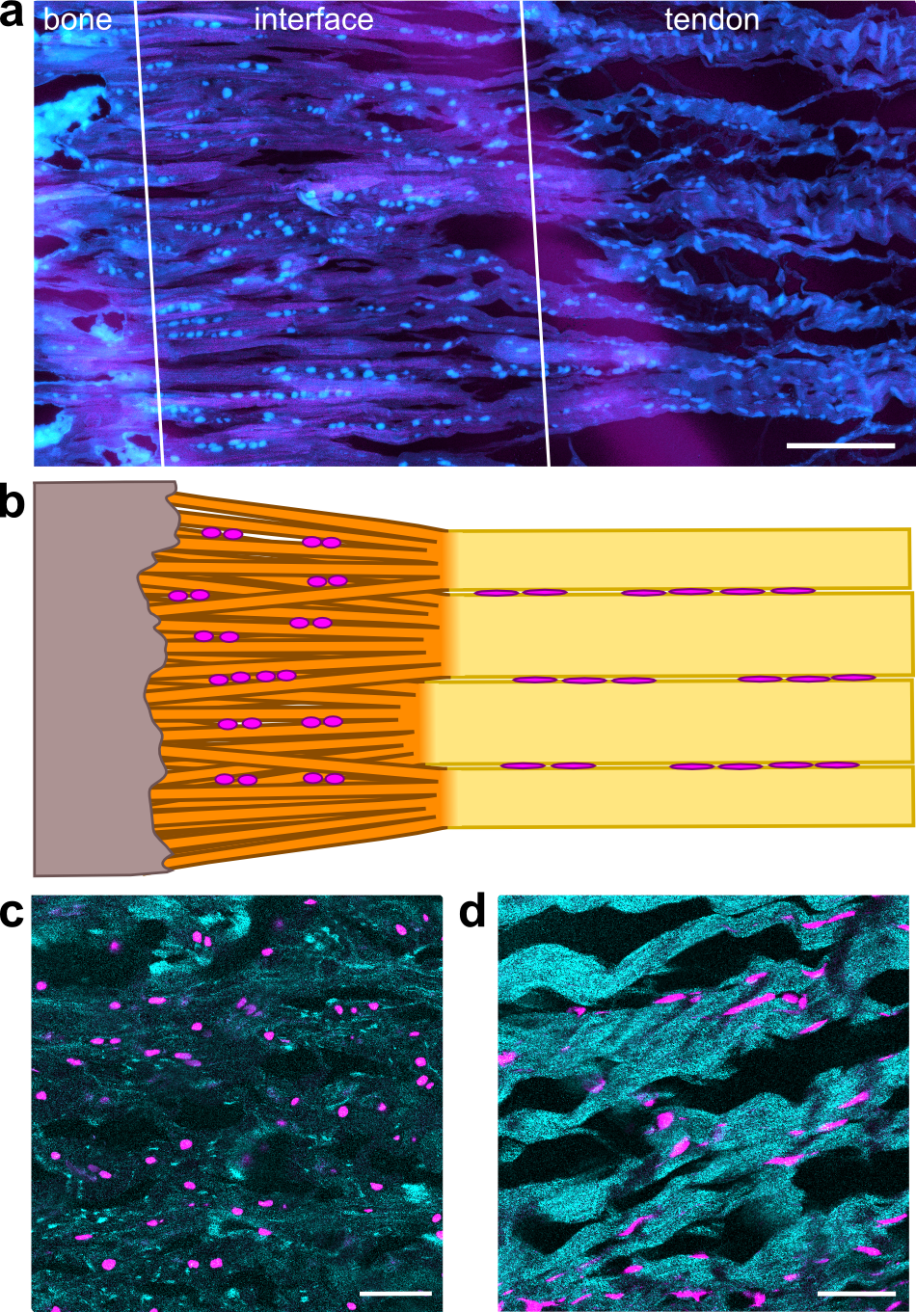
Morphological characteristics of enthesis cells. a, Enthesis cryocut section was stained for cells using SYTO® 13. Cells are depicted cyan, confocal reflection is depicted magenta. Scale bar corresponds to 150 μm. b, Scheme of cell arrangement observed at the enthesis. c, Cells within the interface are round shaped and are often arranged in pairs. Scale bar corresponds 50 μm. d, Cells residing within tendon are longitudinally arranged along the axis of tension in strings between tendon fibers. Scale bar corresponds 50 μm.

Cells respond to the physicochemical cues from their microenvironment[19]. Cells in the tendon-bone interface region reside within a different microenvironment than cells in tendon or bone (Fig 1). In fact, microenvironments of cells within interface region differ geometrically as well as compositionally as discussed in our previous publication[4]. Since gene expression characteristics depend on the stimuli cells are subject to, interface cells are expected to exhibit a different transcriptome than tendon. Thus, for tendon-bone interface tissue engineering, the different microenvironments as well as the different cellular characteristics have to be considered. Here we characterized interface, cartilage, and tendon cells to provide biomarkers for future biomimetic tendon-bone interface tissue engineering strategies.

Morphological differences of cells residing within the Achilles tendon and the interface region of the attachment were observed in confocal microscopy of enthesis cryocut sections stained with fluorescent cell dye Yo-Pro®-1 or SYTO® 13 (Fig 1A, 1C and 1D). Cells residing within the tendon were observed to be arranged in strings of cells that showed an elongated morphology and were aligned in the direction of tendon tension. Cells at the interface were two-dimensionally observed to be round-shaped and to be often arranged pairwise (Fig 1B). The morphology of the cells at the interface resembled morphology of chondrocytes, which are cartilage cells that are arranged in cartilage lacunae (Fig 1C).

**Fig 2:**
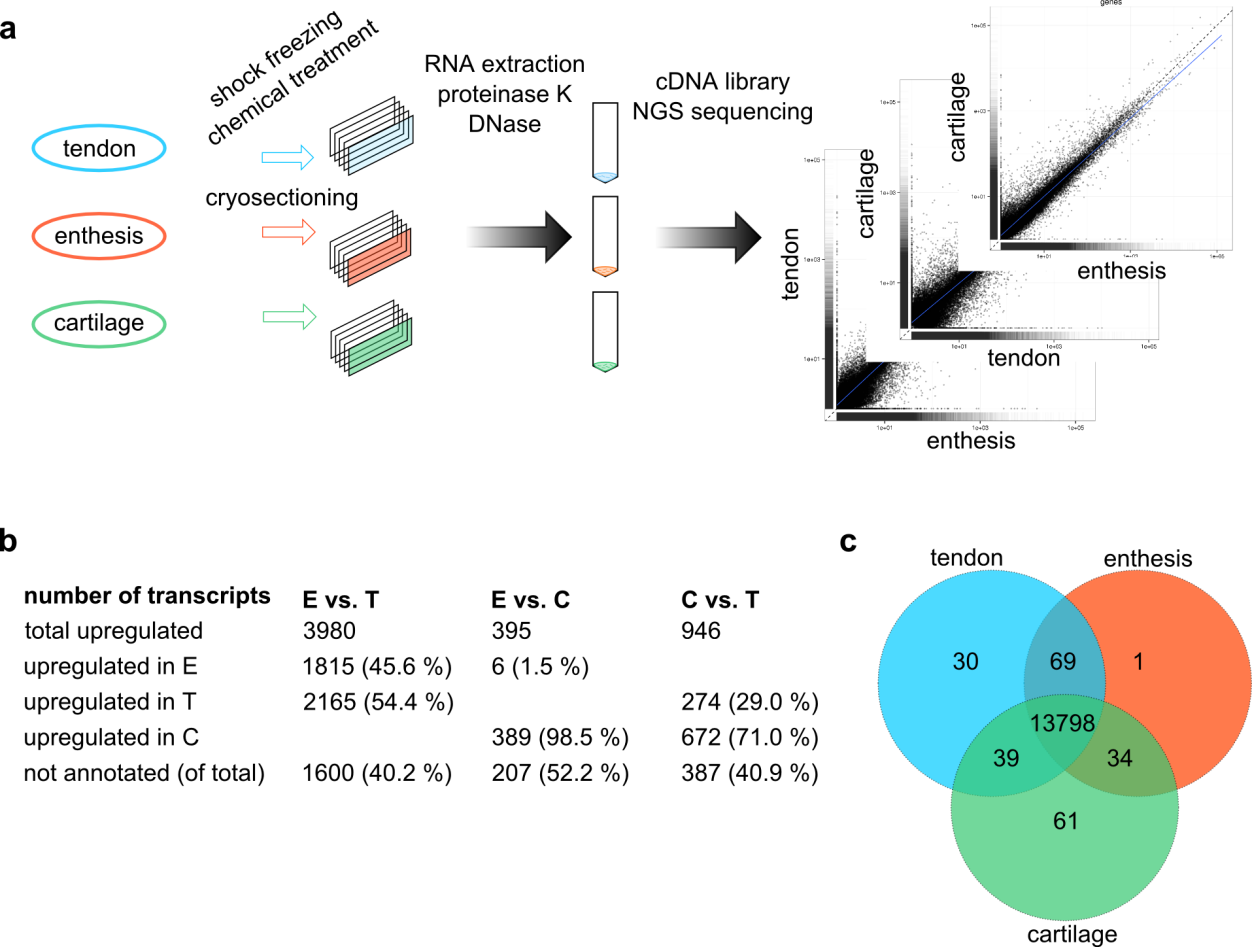
Comparative transcriptome analysis of tendon, enthesis, and cartilage. a, Experimental design of the transcriptomic study. Tendon (blue), interface region (orange), and cartilage (green) samples were excised, shock-frozen in liquid nitrogen, chemically treated to prevent RNA degradation and cryocut sectioned to enhance RNA extraction. High quality RNA was extracted using a refined protocol including proteinase K and DNase digests. cDNA libraries were produced and sequenced using next generation sequencing on an Illumina platform. Transcriptomes of enthesis, tendon, and cartilage were compared to each other. b, Global differences in the transcriptome of enthesis (E), tendon (T), and cartilage (C). c, Venn diagram of genes detected in the three tissues showing the number of overlapping and differentially expressed genes. Only genes that were annotated and had FPKM > 0 were considered, duplicates were not considered twice.

Due to observed morphological differences of cells residing within the interface region compared to tendon cells, cellular differentiation was investigated to identify biomarkers for interface cell differentiation that may result in the observed morphology. In our previous proteome study of interface region and matrix composition, many cartilage-related biomolecules were detected within the interface region[4]. Therefore, interface region cells were not only compared to tendon but also to cartilage cells. Samples were acquired from Achilles tendon enthesis interface region, Achilles tendon, and articular cartilage tissue, shock-frozen, cryocut sectioned, and mRNA was extracted (Fig 2A). Total mRNA samples were reverse transcribed into cDNA libraries for interface region, tendon, and cartilage. cDNA libraries were sequenced by GATC Biotech AG using an Illumina next generation sequencing platform. The raw sequence data of all single end reads was quality checked (QC) and the QC passed sequencing reads were aligned to the pig reference genome Sscrofa10.2. Sscrofa10.2 (database version 87.102) had 21,630 annotated coding genes and 30,585 gene transcripts with 52,372 GENSCAN gene predictions. An average of 88.9% of all QC passed reads were aligned to the reference genome Sus scrofa10.2 (S1 Table). The software Cuffdiff tracked the mapped reads and determined values for fragment per kilobase per million mapped reads (FPKM) for each transcript in all samples indicating the level of expression.

In total, 34468 transcripts were detected in the three analyzed tissues (Fig 2). Of these, 14128 were not functionally annotated meaning that the functions of the proteins that are encoded by the corresponding transcripts were not yet known. Values of FPKM = 0 were observed for 1217 transcripts in the enthesis, 989 transcripts in the tendon, and 382 transcripts in the cartilage indicating that they were either not expressed or below the detection limit with the given sequencing depth. In total, 33251 transcripts with FPKM > 0 were detected in the enthesis, 33479 transcripts with FPKM > 0 were detected in the tendon, and 34083 transcripts with FPKM > 0 were detected in cartilage.

The comparison of the transcriptomes of enthesis and tendon showed 3980 transcripts to be statistically significant differentially expressed in both tissues, of which ~ 46% and ~ 54% were enriched in enthesis and tendon, respectively (Fig 2B). Comparison of enthesis and cartilage transcriptomes identified 395 transcripts to be statistically significant differentially expressed in both tissues, of which ~ 2% were enriched in enthesis and 98% were enriched in cartilage. Comparison of the transcriptomes of cartilage and tendon detected 946 statistically significant differentially expressed transcriptomes in both tissues, of which ~ 29% were enriched in tendon and ~ 71% were enriched in cartilage (Fig 2B).

Lists of all detected transcripts within the three tissues and their corresponding FPKM values were analyzed to identify all candidates with existing gene annotation and FPKM > 0. The identified lists of genes with gene annotation and FPKM > 0 of enthesis, tendon and cartilage were compared using GeneVenn[20]. The venn diagram shows the number of tissue specific genes as well as the number of genes that overlapped between two of the tissues or all tissues (Fig 2C). 13798 genes were expressed in all three tissues. Tendon exhibited 30 tissue specific genes whereas enthesis and cartilage had one tissue specific gene and 61 tissue specific genes, respectively. 39 genes were only expressed in tendon and cartilage, 69 genes in tendon and enthesis, and 34 genes in enthesis and cartilage (Fig 2C).

It has to be noted that the transcriptome difference may be underestimated since a sample preparation precision limitation leads to tendon residues in interface samples. Interface region and tendon are not distinguishable by eye and have to be labeled to be identified. Since fresh tissue has to be used for the transcriptome analysis, a labeling prior to dissection is not eligible. The interface region of the porcine Achilles tendon enthesis spans ~ 500 μm[4]. Since the borders of the interface region have to be visually approximated, tendon residuals may remain within the interface region samples. The differences of tendon and enthesis transcriptome and enthesis and cartilage transcriptome are thus possibly larger than resolved here.

Of the 21640 porcine genes and 26487 known gene transcripts[21], 13976 genes were detected in the three tissues and successfully annotated. The identified lists of genes that were only expressed in one of the three tissues (Fig 2C; 30 genes for tendon, 1 gene for enthesis, and 61 genes for cartilage) were analyzed using PANTHER gene ontology analysis for statistical overrepresentation of biological processes and reactome pathways. The genes expressed in cartilage only showed overrepresentation of reactome pathways acyl chain remodeling of phosphatidylinositol and phosphatidylserine. No overrepresented reactome pathways were identified for the lists of genes only detected in tendon and enthesis. None of the lists of genes that were only expressed in tendon, enthesis, or cartilage showed overrepresented biological processes.

GO analysis of the list of genes that were detected in all three tissues showed overrepresentation of biological processes such as DNA repair, cell component biogenesis, vesicle-mediated transport and underrepresentation of biological processes such as blood circulation, natural killer cell activation, G-protein coupled receptor signaling, and sensory perception of smell. Further, reactome pathways RHO GTPase effectors, processing of capped intron-containing pre-mRNA, transcriptional regulation by TP53, M phase, membrane trafficking, asparagine n-linked glycosylation, and cellular response to stress were overrepresented. The transcriptomes of enthesis and tendon, enthesis and cartilage, as well as cartilage and tendon were compared to identify similarities and differences in the expression patterns of the cells residing within enthesis, tendon, and cartilage.

### Transcriptome comparison of enthesis and tendon

**Fig 3:**
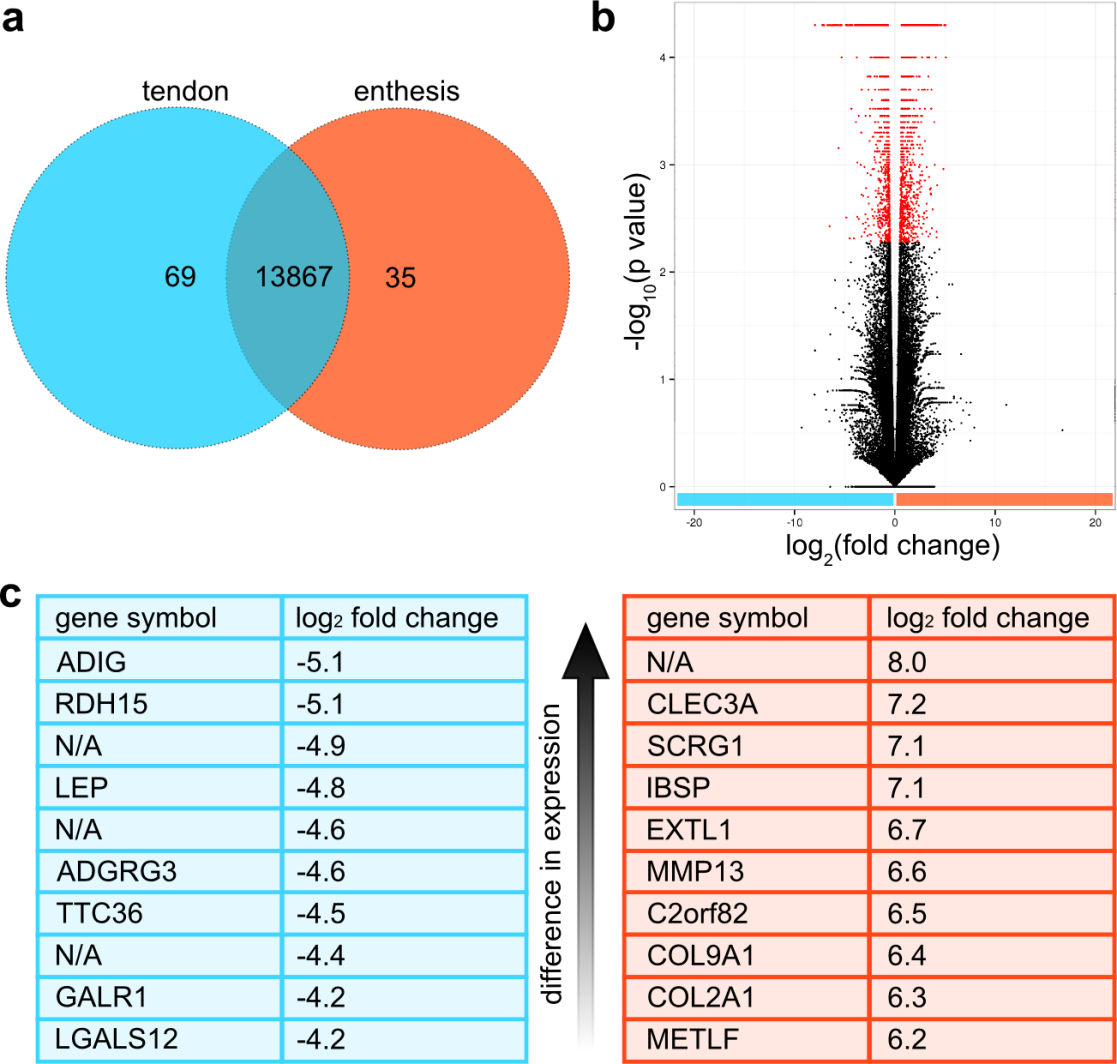
Comparative transcriptome analysis of enthesis and tendon. a, Venn diagram of differences between enthesis and tendon transcriptomes depicts the number of annotated genes that are only present in tendon (69), enthesis (35) or present in both tissues (13867). b, Volcano plot of all differentially enriched transcripts detected in the transcriptomes of tendon and enthesis. Statistically significant enriched genes were marked red. c, Top 10 genes with highest gene expression difference in tendon (blue) or enthesis (orange).

Transcriptome comparison of enthesis cells and tendon cells showed differential expression levels in the two tissues (Fig 3A). For the 34468 transcripts that were detected in total in the two tissues, 13971 corresponding annotated genes were identified that encode for the transcripts. The large gap in the numbers may derive from alternative splicing mechanisms meaning that several alternative spliced transcripts can be encoded by the same gene. Furthermore, since the Sus scrofa genome is not fully annotated, information can get lost due to the inability of allocating certain transcripts to genes. Numerous predicted protein-coding loci have still no functional annotation and there is even less information about the function of many non-protein-coding genes[22].

Of the 13971 identified genes, 13867 were expressed in both enthesis and tendon, 69 genes were detected only in tendon, and 35 genes only in enthesis. GO analysis of the 69 genes detected in tendon showed an overrepresentation of the reactome pathway NCAM1 interactions and the GO molecular function complete for extracellular matrix structural constituents. GO analysis of the 35 genes detected in enthesis only did not identify any overrepresented processes or pathways.

Of all 34468 detected transcripts, 3980 transcripts were identified that were differentially expressed in the two tissues, meaning that they occurred in both tissues, but were expressed in statistically significant different levels (Fig 3B and 3C). 1600 transcripts of the 3980 transcripts that were either enriched in enthesis or tendon were not functionally annotated, so the function remained unknown.

Enthesis tissue compared to tendon showed 1815 statistically significant enriched transcripts in total and 733 of these transcripts were statistically significant at least twofold enriched. 433 of all transcripts enriched in enthesis were not detected in tendon (FPKM = 0). Tendon tissue compared to enthesis tissue exhibited 2165 statistically significant enriched transcripts in total, of which 1648 were statistically significant at least twofold enriched. 635 of the tendon enriched transcripts were not detected in the enthesis (FPKM = 0).

PANTHER overrepresentation test gene ontology (GO) analysis of biological process complete showed multiple enriched biological processes in enthesis tissue compared to tendon. All genes that were at least twofold enriched were analyzed for overrepresentation of biological processes with Sus scrofa as reference list. Overrepresented biological processes included proteoglycan biosynthesis, chondrocyte differentiation, collagen fibril organization, cartilage development, ADP metabolism, stem cell proliferation, morphogenesis of a branching structure, connective tissue development, ossification, ECM organization, angiogenesis, and skeletal development. A GO analysis was also performed for overrepresented reactome pathways and the identified pathways included Abl in Robo-Slit signaling, chondroitin sulfate biosynthesis, glycolysis, collagen biosynthesis, assembly of collagen fibrils, ECM proteoglycans, ECM organization, and integrin cell surface interactions.

GO analysis of the genes at least twofold enriched in tendon identified multiple overrepresented biological processes. The overrepresented biological processes included interferon-gamma production, immune response, leukocyte cell-cell adhesion, lymphocyte activation, cytokine production, fatty acid metabolism, cell migration, chemotaxis, regulation of locomotion, amongst many others. The overrepresented reactome pathways included rhodopsin-like receptors, G protein-coupled receptors signaling and immune system.

The top 10 genes each with statistically significant highest and lowest log_2_ fold change and thus most distinct enrichment in enthesis and tendon, respectively, as well as their gene names and functions are listed in Table 1.

**Table 1:** Top 10 genes with highest and lowest log_2_ fold change in enthesis/tendon transcriptome comparison. Gene names and functions derive from GeneCards^®^ and Ensembl.

Enriched in	Gene symbol	Gene name	Function	Log_2_ fold change
**Enthesis**	-	ID: XLOC_025407	uncharacterized	8.0
	CLEC3A	c-type lectin domain family 3 member a	carbohydrate binding	7.2
	SCRG1	stimulator of chondrogenesis 1	can enhance differentiation potential of mesenchymal stem cells	7.1
	IBSP	integrin binding sialoprotein	binds to calcium and hydroxyapatite, mediates cell attachment	7.1
	EXTL1	exostosin like glycosyltransferase 1	glycosyltransferase, involved in chain elongation of heparan sulfate	6.7
	MMP13	matrix metallopeptidase 13	degradation of extracellular matrix such as in tissue remodeling	6.6
	C2orf82	chromosome 2 open reading frame 82	uncharacterized	6.5
	COL9A1	collagen type IX alpha-1 chain	collagen component of cartilage	6.4
	COL2A1	collagen type II alpha-1 chain	fibrillar collagen found in cartilage and the vitreous humor of the eye	6.3
	MELTF	melanotransferrin	iron binding function	6.2
**Tendon**	ADIG	adipogenin	adipocyte differentiatio	-5.1
	RDH16	retinol dehydrogenase 16 (all-trans)	oxidoreductase	-5.1
	ENSSSCG00000030522	HCOP predicted: alcohol dehydrogenase	uncharacterized	-4.9
	LEP	leptin	regulation of energy balance, acts as a growth factor on certain tissues	-4.8
	ENSSSCG00000010992	SAQP7	uncharacterized	-4.6
	ADGRG3	adhesion G protein-coupled receptor G3	orphan receptor	-4.6
	TTC36	tetratricopeptide repeat domain 36	uncharacterized	-4.5
	ENSSSCG00000009699	-	uncharacterized	-4.4
	GALR1	galanin receptor 1	interaction with G-protein-coupled receptors	-4.2
	LGALS12	galectin 12	beta-galactoside-binding protein	-4.2

Transcription factors and growth factors play an important role in regulation of transcription and differentiation of cells. Generally, transcription factors are molecules that bind to DNA, either directly or indirectly, and regulate their transcription. Growth factors are molecules that interact with other molecules to influence cellular behavior such as proliferation and differentiation. Due to the importance of transcription factors and growth factors for the biological function of cells, the enriched gene lists were analyzed with regard to identification of expressed transcription factors and growth factors using DAVID bioinformatics resources 6.8. Functional Annotation Tool.

**Fig 4:**
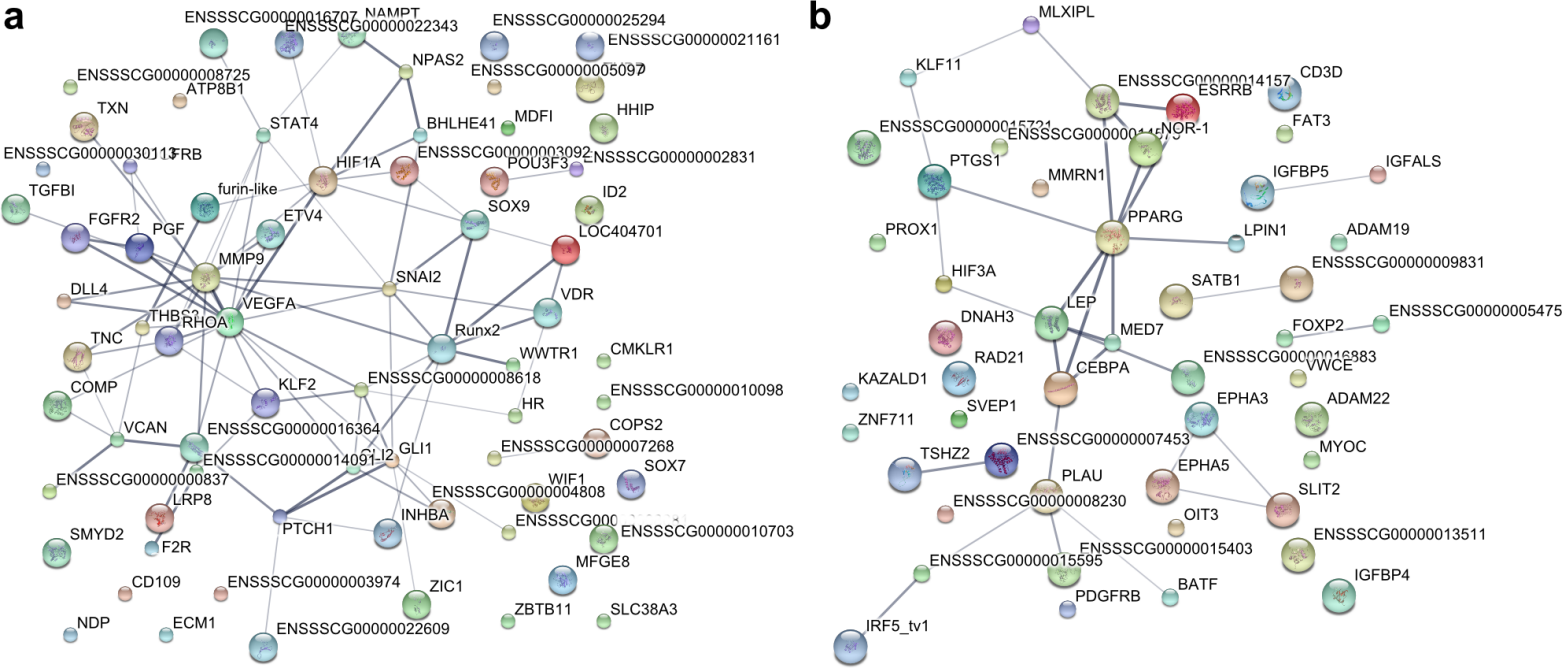
**Protein-protein interaction networks of transcription factors and growth factors enriched in enthesis (a) or tendon (b).** Protein-protein interactions were predicted using the database Search Tool for the Retrieval of Interacting Genes/Proteins (STRING). Line thickness indicates the strength of data support for protein-protein interaction.

In the list of genes enriched in enthesis compared to tendon, 53 candidates with predicted transcription factor activity were identified as well as 23 candidates with predicted growth factor domains. All identified candidates with transcription factor or growth factor activity are listed in the Supplement (S2 Table). The protein-protein interaction network of the identified enthesis transcription factor and growth factor candidates was analyzed using the database Search Tool for the Retrieval of Interacting Genes/Proteins (STRING) of the European Molecular Biology Laboratory (Fig 4A). Interestingly, the two transcription factors RUNX2 and SOX9, which are well-known in musculoskeletal tissues, were identified as nodes within the network.

Additionally, the list of genes enriched in tendon was analyzed for candidates with growth factor or transcription factor activity. 32 candidates with transcription factor activity and 27 candidates with growth factor-like domains were identified within tendon. All identified candidates as well as the respective GO terms of tendon are listed the Supplement (S3 Table). The protein-protein interaction network of the identified candidates with transcription factor or growth factor activity was analyzed using STRING database and several nodes were identified within the network (Fig 4B) such as plasminogen activator (PLAU), CCAAT/enhancer-binding protein alpha (CEBPA), peroxisome proliferator-activated receptor gamma (PPARG), neuron-derived orphan receptor 1 (NOR-1), estrogen related receptor beta (ESRRB).

### Transcriptome comparison of enthesis and cartilage

**Fig 5:**
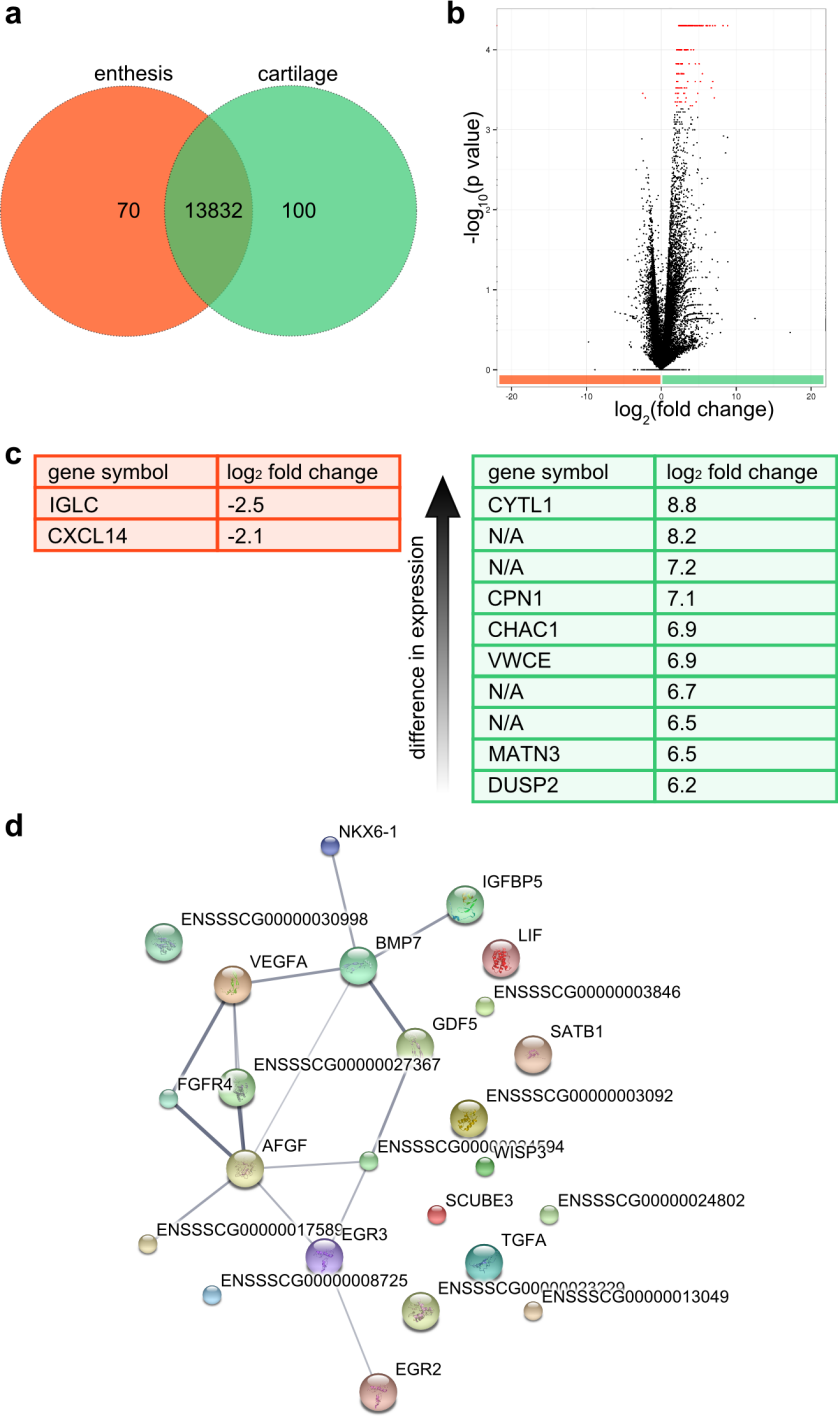
Comparative transcriptome analysis of enthesis and cartilage. a, Venn diagram depicts the number of annotated genes that were only present in enthesis (70) or cartilage (100) or present in both tissues (13832). b, Volcano plot of all differentially enriched genes detected in the transcriptomes of enthesis (orange) and cartilage (green). Statistically significant enriched genes were marked red. c, List of genes with highest enrichment of gene expression in enthesis (orange) or cartilage (green). d, Protein-protein interaction network for transcription factors and growth factors enriched in cartilage compared to enthesis. Protein-protein interactions were predicted using the database Search Tool for the Retrieval of Interacting Genes/Proteins (STRING). Line thickness indicated strength of data support for protein-protein interaction.

Our previous proteome study[4] identified several cartilage-related molecules in the interface region. Therefore, interface region cells were not only compared to tendon cells but also to cartilage cells to identify differences and similarities of cartilage cell expression patterns. Identification of similarities in enthesis and cartilage gene expression may be useful for transferring tissue engineering strategies previously used in more established cartilage tissue engineering to enthesis tissue engineering. Of all detected transcripts in enthesis and cartilage, 14002 were matched to annotated genes and analyzed using GeneVenn[20] (Fig 5A). Of all annotated genes, expression of 13832 genes was detected in both enthesis and cartilage, expression of 70 genes was detected only in enthesis and expression of 100 genes was detected only in cartilage.

Panther GO analysis detected that genes related to the biological process of negative cell cycle regulation were overrepresented in the list of 70 genes that were only expressed in the enthesis. In the list of genes that was only detected in cartilage, an overrepresentation of reactome pathways was observed; hydrolysis of lysophosphatidylcholine and acyl chain remodeling of several phospholipids.

Transcriptome comparison of enthesis and cartilage cells revealed a strong overlap between the two transcriptomes (Fig 5B). Only 6 transcripts were statistically significant enriched in the enthesis cells compared to cartilage cells of which 4 transcripts were not functionally annotated (Fig 5C). The same 4 transcripts that were not functionally annotated further had a value of FPKM = 0 in the cartilage tissue meaning that they were below the detection threshold. In total, 389 transcripts were enriched in cartilage compared to enthesis and were all at least twofold enriched. 203 of these transcripts were not functionally annotated. In total, 197 transcripts that were enriched in cartilage were not detected in the enthesis tissue (FPKM = 0). The top 10 genes with highest and lowest log_2_ fold change and thus enrichment in enthesis or cartilage, respectively, were listed in Table 2.

**Table 2:** Top 10 genes with highest and lowest log_2_ fold change in enthesis/cartilage transcriptome comparison. Gene names and functions derive from GeneCards^®^ and Ensembl.

Enriched in	Gene symbol	Gene name	Function	Log_2_ fold change
**Enthesis**	IGLC	immunoglobulin lambda constant	antigen binding	2.5
	CXCL14	C-X-C motif chemokine ligand 14	cytokine	2.1
	-	XLOC_012195	unknown	∞
	-	XLOC_041171	unknown	∞
	-	XLOC_041579	unknown	∞
	-	XLOC_041580	unknown	∞
**Cartilage**	CYTL1	cytokine like 1	cytokine-like protein	-8.8
	ENSSSCG00000010546	unknown	unknown	-8.2
	ENSSSCG00000000468	HCOP* predicted: WNT inhibitory factor 1	prevents Wnt signalling	-7.2
	CPN1	carboxypeptidase N subunit 1	plasma metallo-protease	-7.1
	CHAC1	chaC glutathione specific γ-glutamylcyclotransferase 1	pro-apoptotic component	-6.9
	VWCE	von willebrand Factor C And EGF Domains	calcium ion binding	-6.9
	ENSSSCG00000023305	HCOP* predicted: metallothionein	binding of heavy metals	-6.7
	ENSSSCG00000030998	HCOP* predicted: WNT inhibitory factor 1 (WIF1)	prevents Wnt signalling	-6.5
	MATN3	matrilin 3	involved in formation of filamentous networks, homeostasis of cartilage/bone	-6.5
	DUSP2	dual specificity phosphatase 2	phosphatase, inactivates ERK1/2	-6.2

* HCOP: HGNC Comparison of Orthology Predictions

The lists of genes that were at least twofold enriched were analyzed using PANTHER GO analysis for overrepresented biological processes and reactome pathways. No overrepresented processes nor reactome pathways were detected in the enthesis. In cartilage, multiple overrepresented biological processes were identified such as chondrocyte differentiation, cartilage development, angiogenesis, morphogenesis of branching epithelium, regulation of MAPK cascade, cell differentiation, developmental process, phosphate metabolism, tissue development, multicellular organismal development, regulation of cell proliferation. The analysis of GO reactome pathways detected acyl chain remodeling of phosphatidylinositol and phosphatidylserine as overrepresented.

The list of enriched genes in cartilage compared to enthesis was screened to identify candidates with transcription factor or growth factor activity. 11 candidates with transcription factor activity were identified as well as 13 candidates with growth factor activity (S4 Table). Candidates with transcription factor or growth factor activity were classified for protein-protein interaction using STRING database. Bone morphogenetic protein 7 (BMP7), vascular endothelial growth factor A (VEGFA) and fibroblast growth factor 1 (AFGF) were identified as nodes within the protein network (Fig 5D). BMP7 plays a role in skeletal development involving the differentiation of mesenchymal cells towards osteoblasts and chondrocytes[23].

### Transcriptome comparison of cartilage and tendon

**Fig 6:**
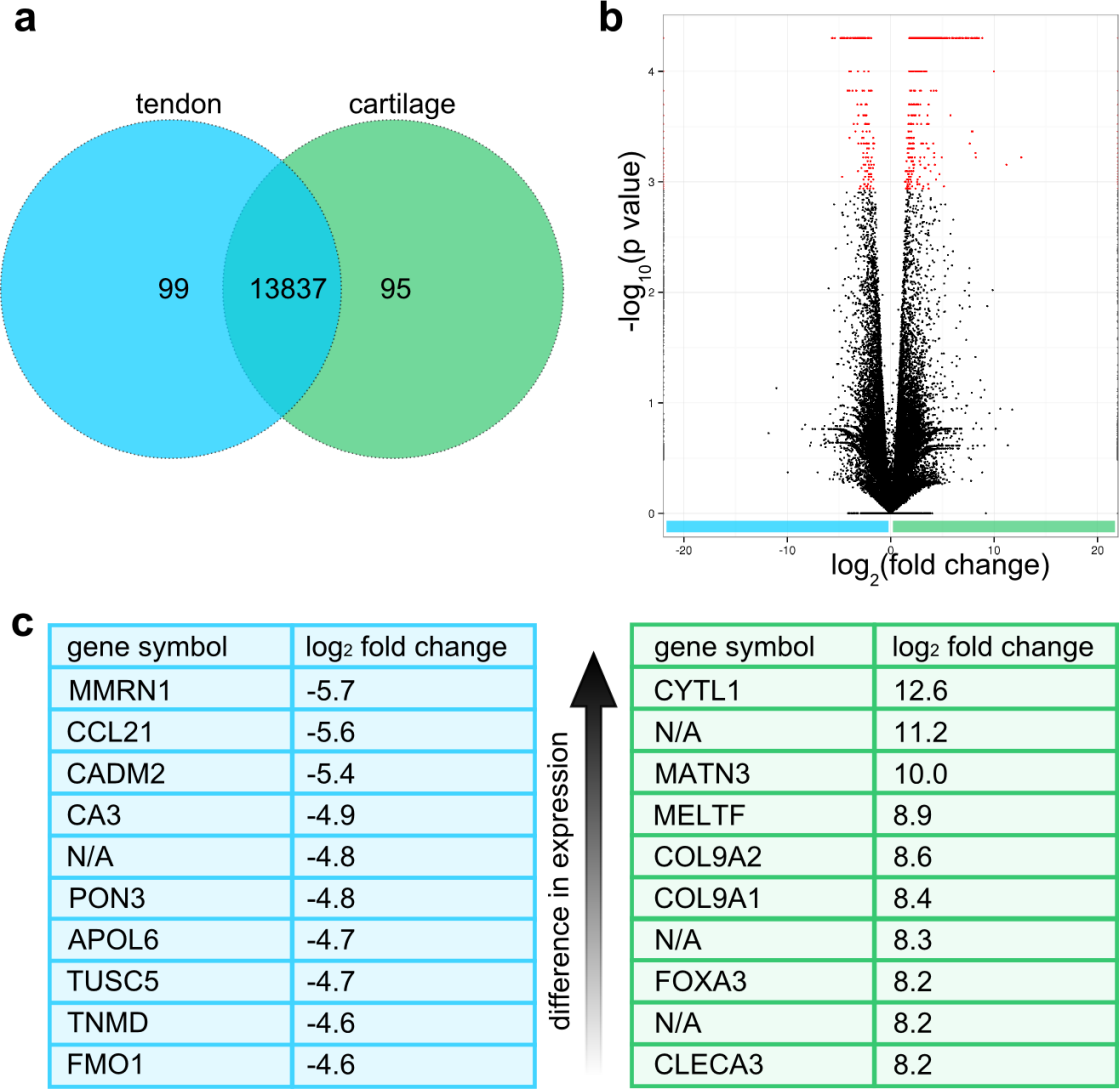
Comparative transcriptome analysis of tendon and cartilage. a, Venn diagram depicts the number of annotated genes that are only expressed in tendon (99) or cartilage (95) or expressed in both tissues (13837). b, Volcano plot of all differentially enriched transcripts detected in the transcriptomes of tendon (blue) and cartilage (green). Statistically significant enriched genes were marked red. c, Top 10 genes with highest enrichment in tendon (blue) or cartilage (green).

For all detected transcripts in cartilage and tendon, 14031 corresponding annotated genes were matched. 13837 expressed and annotated genes were identified in both tendon and cartilage using GeneVenn (Fig 6A). 99 genes were only expressed in tendon. Panther GO analysis did not identify any overrepresented processes or pathways in the set of genes that was solely expressed in tendon using Sus scrofa as reference genome. 95 genes were only expressed in cartilage. The reactome pathway acyl chain remodeling of phosphatidylinositol was overrepresented in the list of genes that were only expressed in cartilage.

Comparison of cartilage and tendon transcriptomes identified 946 differentially expressed transcripts (Fig 6B and 6C). Of these, 672 transcripts were at least twofold enriched in cartilage (~ 71%) and 274 transcripts were at least twofold enriched in tendon (~ 29%). 282 transcripts that were detected in cartilage were not detected in tendon and 105 transcripts that were detected in tendon were not detected in cartilage (FPKM = 0). 387 of the all enriched transcripts were not annotated (~ 41%). The top 10 genes with highest and lowest log_2_ fold change were listed in Table 3.

**Table 3:** Top 10 genes with highest and lowest log_2_ fold change in cartilage/tendon transcriptome comparison. Gene names and functions derive from GeneCards^®^ and Ensembl.

Enriched in	Gene symbol	Gene name	Function	Log_2_ fold change
**Cartilage**	CYTL1	cytokine like 1	cytokine-like protein	12.6
	ENSSSCG00000030998	HCOP predicted: WNT inhibitory factor 1 (WIF1)	prevents Wnt signalling	11.2
	MATN3	matrilin 3	involved in the formation of filamentous networks, involved in development and homeostasis of cartilage and bone	10.0
	MELTF	melanotransferrin	iron binding function	8.9
	COL9A2	collagen type IX alpha-2 chain	collagen component of cartilage; has an attached glycosaminoglycan chain unlike the other type IX alpha chains	8.6
	COL9A1	collagen type IX alpha-1 chain	collagen component of cartilage	8.4
	-	ID: XLOC_025408	unknown	8.3
	FOXA3	forkhead box A3	transcription factor	8.2
	-	ID: XLOC_025406	unknown	8.2
	CLEC3A	c-type lectin domain family 3 member A	carbohydrate binding	8.2
**Tendon**	MMRN1	multimerin 1	carrier protein for platelet factor V	-5.7
	CCL21	C-C motif chemokine ligand 21	CC cytokine	-5.6
	CADM2	cell adhesion molecule 2	cell adhesion molecule	-5.4
	CA3	carbonic anhydrase 3	reversible hydration of carbon dioxide	-4.9
	ENSSSCG00000001844	PLIN	lipid storage	-4.8
	PON3	paraoxonase 3	associates with high-density lipoprotein	-4.8
	APOL6	apolipoprotein L6	lipid binding	-4.7
	TUSC5	tumor suppressor candidate 5	may be involved in fat metabolism	-4.7
	TNMD	tenomodulin	angiogenesis inhibitor	-4.6
	FMO1	flavin containing monooxygenase 1	flavoenzyme	-4.6

GO analysis of enriched genes detected several overrepresented biological processes in cartilage compared to tendon. Proteoglycan biosynthesis, cartilage development, glycosaminoglycan biosynthesis, chondrocyte differentiation, morphogenesis of a branching structure, biomineral tissue development, endochondral bone morphogenesis, bone mineralization, hair cycle, molting cycle, odontogenesis, extracellular matrix organization, stem cell differentiation, amongst others. Further, overrepresented reactome pathways were identified in the cartilage compared to tendon including hedgehog ‘on’ state, glycosaminoglycan metabolism, and extracellular matrix organization.

GO analysis of genes enriched in tendon compared to cartilage identified multiple overrepresented biological processes such as neutrophil/granulocyte chemotaxis, protein kinase B signaling, response to external stimulus, regulation of locomotion, cell migration, regulation of body fluid levels, lipid metabolism, cell adhesion. GO analysis did not detect any overrepresented reactome pathways for the list of genes that was enriched in tendon compared to cartilage.

### Biomarkers for tissue engineering of the tendon-bone interface

**Fig 7:**
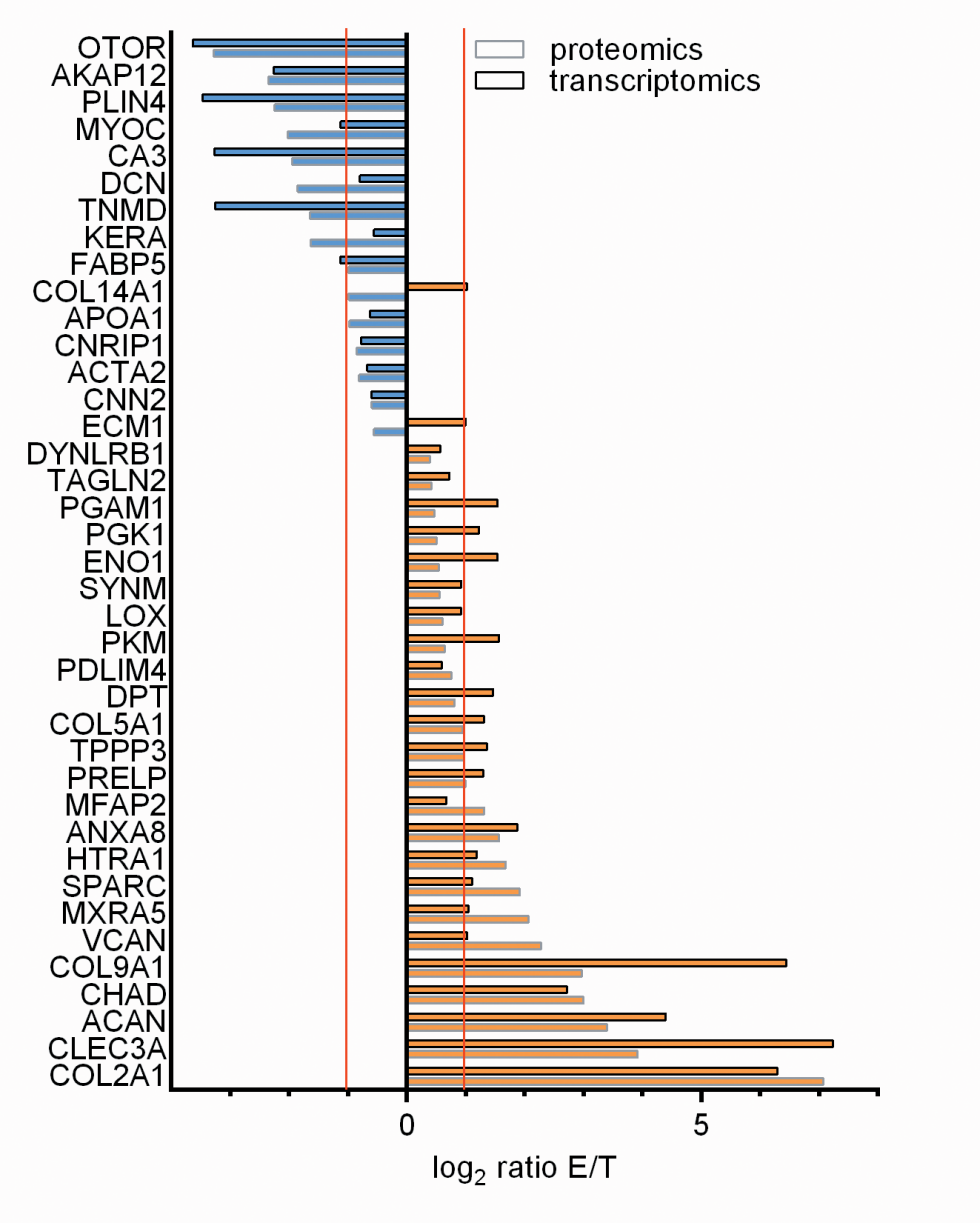
Identified biomarkers of enthesis and tendon. Biomarkers are presented which were statistically significant enriched in both the proteomics and transcriptomics data sets of enthesis (E) or tendon (T). Orange and blue bars represent enrichment in enthesis and tendon, respectively. Gray boxes correspond to enrichment in proteome and black boxes to enrichment in transcriptome. 13 genes were identified as tendon biomarkers (blue) and 24 genes as enthesis biomarkers (orange). Two genes (COL14A1 and ECM1) were identified that were enriched in the tendon proteome and conversely in the enthesis transcriptome.

Strong markers for differentiation of interface cells were identified by comparing the data sets of the proteome and transcriptome studies of tendon and enthesis (Fig 7 and Table 4). Interface cells showed elevated levels of cartilage-related biomarkers in the integrated comparison of proteome and transcriptome of tendon and enthesis. Interestingly, interface cells also showed high similarity with chondrocytes in the transcriptomic comparison of enthesis and cartilage (Fig 5). All genes were regarded as biomarkers that were statistically significant enriched in the proteome and transcriptome data sets of either tendon or enthesis. In total, 39 genes were identified to be enriched in either tendon or enthesis in both data sets of proteome and transcriptome. Fig 7 shows all 39 genes that were differentially expressed in both the proteomics and transcriptomics sets with their corresponding log_2_ ratios.

**Table 4:** Biomarkers that were at least twofold enriched in both transcriptome and proteome. The biomarkers as well as the respective functions and log_2_ ratios are depicted. Log_2_ ratio differences of proteome and transcriptome data sets were calculated for each candidate.

Biomarker	Protein	Function
**Enthesis**		
ACAN	Aggrecan	hyalectan proteoglycan, cartilage-specific core protein
ANXA8	Annexin A8	bone-matrix induced protein
CHAD	chondroadherin	small leucine-rich proteoglycan, interacts with collagen type II
CLEC3A	C-Type Lectin Domain Family 3 Member A; Cartilage derived c-type lectin	cell adhesion
COL2A1	Collagen type II, alpha-1 chain	fibrillar collagen
COL9A1	Collagen type IX, alpha-1 chain	cross-links to surface of type II collagen fibrils
HTRA1	HtrA Serine Peptidase 1	serine protease targeting extracellular matrix proteins such as fibronectin
MXRA5	Matrix-remodelling associated 5	adhesion protein
SPARC	Osteonectin	promotes mineral crystal formation
VCAN	Versican	hyalectan proteoglycan
**Tendon**		
AKAP12	a-kinase anchor protein 12	scaffold protein in signal transduction, binds to protein kinase a
CA3	Carbonic anhydrase 3	metalloenzyme
MYOC	Myocilin	cytoskeletal function
OTOR	Otoraplin	cartilage development suggested
PLIN4	Perilipin-4	coats lipid droplets in adipocytes
TNMD	tenomodulin	Tendon maturation

24 enthesis biomarkers were identified (orange in Fig 7). The identified 24 genes showed enrichment in both proteomics and transcriptomics data sets of the interface region. 13 tendon biomarkers were identified that were enriched in both the tendon transcriptome and proteome (blue in Fig 7). Interestingly, two candidates were identified that were enriched in the tendon proteome, but the enthesis transcriptome. These two genes were COL14A1 and ECM1 and encode two extracellular matrix proteins: collagen type XIV alpha 1 chain and extracellular matrix protein 1, respectively.

Of all enriched biomarker candidates, 16 candidates were at least twofold enriched in both transcriptome and proteome (Table 4). Ten of these genes were enriched in the enthesis and six of these genes were enriched in the tendon. The respective biomarker candidates as well as their gene products’ functions and the corresponding log_2_ ratios were listed in Table 4.

## Discussion

Tissue engineering is a promising and evolving field to tackle the challenge of tissue interface regeneration. Most strategies of tissue engineering involve the use of scaffolds of any sort in combination with cells to biomimic the physiological behavior of the respective tissue. However, in-depth knowledge about the cells present in these tissues and their differential behavior is required to use their full potential for tissue engineering strategies. Tendon cells are relatively well characterized. They are described to be spindle shaped and arranged in long, parallel chains[24]. Tendon cells, namely tenocytes and tenoblasts, are known to comprise ~ 90% of the cells within tendon[24]. The residual ~ 10% of cells in tendon are, for example, chondrocytes that are located at pressure and insertion sites and vascular cells such as capillary endothelial cells[24]. However, relatively little is known about the cells present at the interface region between tendon and bone.

The morphology of interface cells was observed to differ from the morphology of tendon cells in confocal microscopy (Fig 1), so that the transcriptomes of interface cells were compared to tendon cells to identify their differentiation characteristics. Since a previous proteome analysis[4] identified many cartilage-related cells within the interface region, interface cells were not only compared to tendon cells, but also to cartilage cells of the tibial plateau. Identification of similarities between cartilage cells and enthesis cells may be particularly useful for transferring established cartilage tissue engineering strategies to enthesis tissue engineering.

Comparison of enthesis and tendon transcriptomes showed that of 3980 enriched transcripts, ~ 50% were each enriched in enthesis or tendon, indicating that the cells have distinct expression patterns and may undergo different differentiation lineages. The list of genes only expressed in tendon showed overrepresentation of the NCAM1 interaction reactome pathway. NCAM1 is considered to be a cell adhesion mediator[25] and may play a role in the mechanotransduction process that transmits mechanical stimuli onto cells. The list of genes enriched in tendon showed an overrepresentation of biological processes related to immune response, cell migration, and fatty acid metabolism. Biological processes related to fatty acid metabolism were not only identified in the tendon transcriptome, but were also detected in the tendon proteome[4]. This is readily explainable considering interspersed fat deposits in the interfibrillar spaces between tendon fibers. G protein-coupled receptor (GPCR) signaling appears to play an important role in tendon cell signaling, since several reactome pathways related to GPCR were overrepresented. In the interface region, 35 genes were detected that were not found in tendon. Many of these genes have not yet been annotated thus the respective encoded proteins are still unknown. The list of top 10 genes with highest log_2_ fold change in enthesis compared to tendon included two collagens, namely collagen type II alpha-1 and collagen type IX alpha-1. Interestingly, the two collagens were also enriched in the enthesis proteome[4]. The list of top 10 genes also included MMP13 which is known to be involved in degradation of extracellular matrix[26] and may play a role in tissue remodeling processes to adapt to mechanical stresses[27].

The protein-protein interaction network of transcription factors and growth factors enriched in the enthesis identified RUNX2 and SOX9 as nodes within the network. RUNX2 is a transcription factor that is essential to bone development and regulates the differentiation of chondrocytes and osteoblasts[28, 29]. SOX9 is a key regulator of chondrogenesis and has been suggested to mediate differentiation of tenocytes towards the chondrocyte lineage[30]. It has been suggested that, in mice, a scleraxis and SOX9 positive progenitor pool (Scx+/Sox9+) is a unique multipotent cell population that gives rise to tenocytes, ligamentocytes and chondrocytes for the establishment of the chondro-tendinous and ligamentous junction[31] and that expression of Scx and SOX9 may play a role in enthesis development[31, 32]. Moreover, Hedgehog signaling within developing enthesis fibrocartilage cells has also been shown to be required for enthesis development[33].

The list of all genes enriched in enthesis showed enrichment of processes involved in collagen fibril organization and morphogenesis of a branching structure. The expression of these corresponding genes by the cells within the interface region may be a molecular cause for the branched, splayed structures of collagen type II-rich fibers present at the interface region that were elucidated in a previous study[4]. Furthermore, GO analysis showed many enriched biological processes involved in chondrocyte differentiation. For example, SCRG1, stimulator of chondrogenesis and known to enhance differentiation potential of mesenchymal stem cells, was strongly enriched in the interface region. This indicates that cells present at the interface region show close similarity to cartilage chondrocytes which was further elucidated in the transcriptome comparison of enthesis and cartilage.

Comparison of enthesis and cartilage transcriptomes showed an asymmetric distribution of enriched genes. The volcano plot showed (Fig 5B) that multiple genes were enriched in cartilage compared to enthesis, but only very few were enriched in enthesis compared to cartilage. This may indicate that the cells present within the interface region derive from chondrocytes, but either remain in a certain differentiation stage or that they are a “reduced” version of a chondrocyte. Only two of the genes enriched in the enthesis compared to cartilage were annotated, therefore no conclusions can be drawn with regard to gene ontology of biological processes. CXCL14 is one of the genes enriched in enthesis, a cytokine known to inhibit angiogenesis[34]. Previously it has been suggested that there may be inhibitory molecules expressed by enthesis cells due to the avascularity of entheses[35]. CXCL14 may be one of these molecules and play a role in the avascularity of entheses.

The genes enriched in cartilage compared to enthesis showed overrepresented biological processes such as chondrocyte differentiation, angiogenesis, morphogenesis of branching epithelium, and cell differentiation. Interestingly, several processes such as chondrocyte differentiation and cartilage development shown to be overrepresented in enthesis compared to tendon, were also overrepresented in cartilage compared to enthesis. This may indicate that enthesis cells are much more chondrocyte-like than tendon cells, but not as much differentiated within the chondrocyte lineage as cartilage cells. This would be also consistent with the GO biological process of cell differentiation being overrepresented in cartilage cells compared to enthesis cells.

Transcriptome comparison of cartilage and tendon showed symmetric differences as did enthesis and tendon transcriptomes. The top 10 enriched genes in cartilage compared to tendon included cytokine like 1 and matrilin 3, which were also found in the top 10 enriched genes in cartilage compared to enthesis, thus seemingly playing an important role in cartilage. Two collagen type IX chains, namely collagen type IX alpha-1 and collagen type IX alpha-2 were also highly enriched compared to tendon. Collagen type IX alpha-1 was also observed to be enriched in enthesis compared to tendon. Interestingly, CLEC3A was highly elevated in both cartilage and enthesis compared to tendon. CLEC3A is suggested to bind to heparin sulfate proteoglycans on cell surfaces and thus enhancing cell adhesion via integrins[36]. The exact function of this molecule within the matrices of cartilage and enthesis have yet to be elucidated. The top 10 enriched genes in tendon compared to cartilage included several genes involved in lipid binding, storage and metabolism. This is, as previously mentioned, readily explainable considering the distribution of fat deposits within the interfibrillar spaces of tendon fibers. It is to mention that the top 10 genes enriched in tendon compared to enthesis and tendon compared to cartilage completely differ.

Tissue engineering approaches of tendon-bone interfaces rely on deep understanding of the cellular processes occurring at the natural tendon-bone interface. Identification of biomarkers enables the precise characterization of cells cultured on artificial tendon-bone interface scaffolds, such as design-engineered biomaterial scaffolds[5, 37] or decellularized donor scaffolds[10, 11, 38–41] with regard to their differentiation status. Here, strong biomarkers for enthesis cell differentiation were identified by integrating proteomic and transcriptomic data. All candidate genes that were statistically significant at least twofold enriched both on protein level (proteome) and the mRNA level (transcriptome) were considered to be strong markers for enthesis cell differentiation. In total, 39 genes were identified that were enriched on both the transcript level and the protein level. 13 of these genes were enriched in the transcriptome and proteome of tendon and 24 of these genes were enriched in the transcriptome and proteome of the interface region. Interestingly, the remaining two genes showed enrichment in the tendon proteome, but conversely in the enthesis transcriptome. These two candidates were collagen type XIV alpha-1 chain and extracellular matrix protein 1. Collagen type XIV is a FACIT collagen that interacts with collagen fibers, possibly through interaction with small proteoglycans such as decorin and fibromodulin[42]. Extracellular matrix protein 1 interacts with a variety of extracellular proteins and is suggested to be involved in various functions[43] such as endochondral bone formation and angiogenesis. The observed difference between upregulation in transcriptome and proteome may derive from changing mechanical stimuli that the cells undergo. The half-life of proteins within the extracellular matrix differs from the half-life of the corresponding mRNA[44]. Therefore, it is conceivable that the observed difference in enrichment may derive from an adaptational process in which the cells respond to an altered demand.

In the integrated analysis of proteome and transcriptome, 16 genes identified that were statistically significant at least twofold enriched on both the protein and transcript level in tendon or enthesis. Six of these genes were enriched in the tendon, ten of these genes were enriched in the enthesis. Within the enthesis, cartilage-related biomolecules were identified such as aggrecan, chondroadherin, collagen type II, and versican. Interestingly, several markers of terminal hypertrophic chondrocytes were detected in the cells within the interface such as runt-related transcription factor 2 (RUNX2), integrin binding sialoprotein (IBSP), and matrix metallopeptidase 13 (MMP13)[28].

Since fresh tissue had to be used for transcriptome analysis, a labeling of the ~ 500 μm spanning interface region prior to dissection was not eligible. Thus, enthesis sample preparation was limited by visual approximation of the interface region. Since tendon residuals may remain within the enthesis samples, the difference in expression may be even larger than resolved here.

Conclusively, several biomarkers were identified that can be used to characterize cells on tendon-bone interface scaffolds. This is especially useful with regard to most scientists using mesenchymal stem cells to seed tendon-bone interface scaffolds. Mesenchymal stem cells have the potential to differentiate towards fibroblasts, adipoblasts, chondroblasts, myoblasts, and osteoblasts depending on the stimuli[45]. The combination of biomarkers that has been identified in this study is useful as first comparative benchmark to study whether the in vitro expression patterns of cells cultured on a scaffold correspond to physiologically relevant expression patterns of enthesis cells. Further, by seeding mesenchymal stem cells onto decellularized donor scaffolds, it could be investigated whether the cell microenvironment itself is sufficient to trigger cells towards a given differentiation lineage or whether extensive treatment with growth factors is needed.

## Supporting information

S1 Table Summary of sequence alignment to the genome of Sus scrofa. Number of reads mapped to the porcine reference genome Sus scrofa10.2.
(DOCX)

S2 Table Enthesis transcription factors and growth factors. Candidates with transcription factor or growth factor activity were identified within the transcripts that were enriched in the enthesis compared to tendon.
(DOCX)

S3 Table Tendon transcription factors and growth factors. Candidates with transcription factor or growth factor activity were identified within the transcripts that were enriched in tendon compared to enthesis.
(DOCX)

S4 Table Cartilage transcription factors and growth factors. Candidates with transcription factor or growth factor activity were identified within the transcripts that were enriched in cartilage compared to enthesis.
(DOCX)
