# Supplementary material for: Biomarkers for tissue engineering of the tendon-bone interface
Source: PLoS One. 2018 Jan 3;13(1):e0189668. doi: 10.1371/journal.pone.0189668 (PMC5751986; doi:10.1371/journal.pone.0189668)
Supplement: S1 Table — Number of reads mapped to the porcine reference genome Sus scrofa10.2. (DOCX) [file pone.0189668.s001.docx]

**Biomarkers for tissue engineering of the tendon-bone interface**

Lara A. Kuntz^1,2,*^, Leone Rossetti^2^, Elena Kunold^3^, Andreas Schmitt^1^, Ruediger von Eisenhart-Rothe^1^, Andreas R. Bausch^2^, Rainer H. Burgkart^1,*^

^1^ Klinik für Orthopädie und Sportorthopädie, Klinikum rechts der Isar, Technische Universität München, D-81675 München, Germany

^2^ Lehrstuhl für Zellbiophysik, Technische Universität München, D-85748 Garching, Germany.

^3^ Center for Integrated Protein Science (CIPSM), Department of Chemistry, Technische Universität München, D-85747 Garching, Germany.

*to whom correspondence should be addressed: [kuntz@tum.de](mailto:kuntz@tum.de) and [burgkart@tum.de](mailto:burgkart@tum.de)

# Supplement

## S1 Table

Table 1: Summary of sequence alignment to the genome of Sus Scrofa. Number of reads mapped to the porcine reference genome Sus scrofa10.2.

| **Sample** | **QC passed reads** | **Mapped Reads** | **% Mapped** |
| --- | --- | --- | --- |
| Enthesis Pool 1 | 95,101,732 | 84,440,834 | 88.79 |
| Enthesis Pool 2 | 95,101,732 | 84,440,834 | 88.79 |
| Enthesis Pool 3 | 30,955,077 | 27,599,523 | 89.16 |
| Tendon Pool 1 | 37,869,610 | 33,618,568 | 88.77 |
| Tendon Pool 2 | 53,789,951 | 48,086,845 | 89.40 |
| Tendon Pool 3 | 31,952,065 | 28,443,958 | 89.02 |
| Cartilage Pool 1 | 95,101,732 | 84,440,834 | 88.79 |
| Cartilage Pool 2 | 60,972,484 | 54,047,466 | 88.64 |
