# Supplementary material for: Biomarkers for tissue engineering of the tendon-bone interface
Source: PLoS One. 2018 Jan 3;13(1):e0189668. doi: 10.1371/journal.pone.0189668 (PMC5751986; doi:10.1371/journal.pone.0189668)
Supplement: S2 Table — Candidates with transcription factor or growth factor activity were identified within the transcripts that were enriched in the enthesis compared to tendon. (DOCX) [file pone.0189668.s002.docx]

**Biomarkers for tissue engineering of the tendon-bone interface**

Lara A. Kuntz^1,2,*^, Leone Rossetti^2^, Elena Kunold^3^, Andreas Schmitt^1^, Ruediger von Eisenhart-Rothe^1^, Andreas R. Bausch^2^, Rainer H. Burgkart^1,*^

^1^ Klinik für Orthopädie und Sportorthopädie, Klinikum rechts der Isar, Technische Universität München, D-81675 München, Germany

^2^ Lehrstuhl für Zellbiophysik, Technische Universität München, D-85748 Garching, Germany.

^3^ Center for Integrated Protein Science (CIPSM), Department of Chemistry, Technische Universität München, D-85747 Garching, Germany.

*to whom correspondence should be addressed: [kuntz@tum.de](mailto:kuntz@tum.de) and [burgkart@tum.de](mailto:burgkart@tum.de)

# Supplement

## Identified transcription factors and growth factors

### S2 Table: Enthesis transcription factors and growth factors

Candidates with transcription factor or growth factor activity were identified within the transcripts that were enriched in the enthesis compared to tendon.

Table S2: Candidates with transcription factor or growth factor activity enriched in enthesis compared to tendon.

| **ID** | **Gene Name** | **GO molecular function terms (selected) or InterPro domain** |
| --- | --- | --- |
| **Transcription factors** |  |  |
| ENSSSCG00000003974 | Cbp/p300 interacting transactivator with Glu/Asp rich carboxy-terminal domain 4 (CITED4) | GO:0000988~transcription factor activity |
| ENSSSCG00000022096 | POU class 3 homeobox 3 (POU3F3) | GO:0001071~nucleic acid binding transcription factor activity |
| ENSSSCG00000011704 | WW domain containing transcription regulator 1 (WWTR1) | GO:0000988~transcription factor activity |
| ENSSSCG00000025551 | runt-related transcription factor 2, RUNX2 (LOC100737965) |  |
| ENSSSCG00000000443 | DNA-binding GLI family zinc finger 1 (GLI1) | GO:0003676~nucleic acid binding |
| ATP8B1 | ATPase phospholipid transporting 8B1 | GO:0045892~negative regulation of transcription |
| BARX1 | BARX homeobox 1 | GO:0006357~regulation of transcription from RNA polymerase II promoter |
| CKS2 | CDC28 protein kinase regulatory subunit 2 | GO:0045893~positive regulation of transcription |
| COPS2 | COP9 signalosome subunit 2 | GO:0045892~negative regulation of transcription |
| E2F1 | E2F transcription factor 1 | GO:0000122~negative regulation of transcription from RNA polymerase II promoter |
| ETV4 | ETS variant 4 | GO:0006357~regulation of transcription from RNA polymerase II promoter |
| F2RL1 | F2R like trypsin receptor 1 | GO:0000187~activation of MAPK activity |
| GLI2 | GLI family zinc finger 2 | GO:0000122~negative regulation of transcription from RNA polymerase II promote |
| KLF4 | Kruppel like factor 4 | GO:0032088~negative regulation of NF-kappaB transcription factor activity |
| MDFI | MyoD family inhibitor | GO:0045892~negative regulation of transcription |
| NDP | NDP, norrin cystine knot growth factor | GO:0045893~positive regulation of transcription |
| RAB7B | RAB7B, member RAS oncogene family(RAB7B) | GO:0051092~positive regulation of NF-kappaB transcription factor activity |
| SMYD2 | SET and MYND domain containing 2 | GO:0000122~negative regulation of transcription from RNA polymerase II promoter |
| SOX7 | SRY-box 7 | GO:0001706~endoderm formation |
| SOX9 | SRY-box 9 | GO:0001502~cartilage condensation |
| SP7 | Sp7 transcription factor | GO:0001649~osteoblast differentiation |
| THAP7 | THAP domain containing 7 | GO:0045892~negative regulation of transcriptio |
| WWP2 | WW domain containing E3 ubiquitin protein ligase 2 | GO:0043433~negative regulation of sequence-specific DNA binding transcription factor activity |
| ZIC1 | Zic family member 1 | GO:0007389~pattern specification process, |
| AIRE | autoimmune regulator | GO:0045944~positive regulation of transcription from RNA polymerase II promoter, |
| BHLHE41 | basic helix-loop-helix family member e41 | GO:0006351~transcription |
| CMKLR1 | chemerin chemokine-like receptor 1 | GO:0045600~positive regulation of fat cell differentiation |
| F2R | coagulation factor II thrombin receptor | GO:0000187~activation of MAPK activity |
| CYTL1 | cytokine like 1 | GO:0002062~chondrocyte differentiation |
| DLL4 | delta like canonical Notch ligand 4 | GO:0000122~negative regulation of transcription from RNA polymerase II promoter |
| ENO1 | enolase 1 | GO:0045892~negative regulation of transcription |
| ECM1 | extracellular matrix protein 1 | GO:0006357~regulation of transcription from RNA polymerase II promoter |
| FOXA3 | forkhead box A3 | GO:0045944~positive regulation of transcription from RNA polymerase II promoter, |
| GREM1 | gremlin 1, DAN family BMP antagonist | GO:0051092~positive regulation of NF-kappaB transcription factor activity |
| HR | hair growth associated | GO:0006355~regulation of transcription |
| HOXA1 | homeobox A1 | GO:0006355~regulation of transcription |
| HIF1A | hypoxia inducible factor 1, alpha subunit (basic helix-loop-helix transcription factor) | GO:0051216~cartilage development |
| INHBA | inhibin beta A subunit | GO:0006357~regulation of transcription from RNA polymerase II promoter |
| ID2 | inhibitor of DNA binding 2, HLH protein | GO:0000122~negative regulation of transcription from RNA polymerase II promote |
| IRX3 | iroquois homeobox 3 | GO:0006355~regulation of transcription, DNA-templated |
| NPAS2 | neuronal PAS domain protein 2 | GO:0006351~transcription |
| NAMPT | nicotinamide phosphoribosyltransferase | GO:0045944~positive regulation of transcription from RNA polymerase II promote |
| PTCH1 | patched 1 | GO:0000122~negative regulation of transcription from RNA polymerase II promoter |
| RHOA | ras homolog family member A | GO:0043124~negative regulation of I-kappaB kinase/NF-kappaB signaling |
| RGCC | regulator of cell cycle | GO:0045944~positive regulation of transcription from RNA polymerase II promoter |
| STAT4 | signal transducer and activator of transcription 4 | GO:0006351~transcription |
| SNAPC1 | small nuclear RNA activating complex polypeptide 1 | GO:0042795~snRNA transcription from RNA polymerase II promoter |
| SNAI2 | snail family transcriptional repressor 2 | GO:0003676~nucleic acid binding |
| SLC38A3 | solute carrier family 38 member 3 | GO:0061402~positive regulation of transcription from RNA polymerase II promoter in response to acidic pH |
| TXN | thioredoxin | GO:0000122~negative regulation of transcription from RNA polymerase II promoter |
| MYCN | v-myc avian myelocytomatosis viral oncogene neuroblastoma derived homolog | GO:0045944~positive regulation of transcription from RNA polymerase II promoter |
| VDR | vitamin D (1,25- dihydroxyvitamin D3) receptor | GO:0000122~negative regulation of transcription from RNA polymerase II promoter |
| ZBTB11 | zinc finger and BTB domain containing 11 | GO:0006355~regulation of transcription |
| **Growth factor activity** |  |  |
| ENSSSCG00000030998 | WNT inhibitory factor 1 (WIF1) | IPR000742:Epidermal growth factor-like domain |
| ENSSSCG00000010698 | fibroblast growth factor receptor 2 (FGFR2) | GO:0000166~nucleotide binding, |
| ENSSSCG00000005494 | tenascin C (TNC) | IPR000742:Epidermal growth factor-like domain |
| ENSSSCG00000001695 | vascular endothelial growth factor A (VEGFA). | GO:0005102~receptor binding |
| CD109 | CD109 molecule | GO:0030512~negative regulation of transforming growth factor beta receptor signaling pathway |
| HTRA1 | HtrA serine peptidase 1 | GO:0030512~negative regulation of transforming growth factor beta receptor signaling pathway |
| LRP8 | LDL receptor related protein 8 | IPR000742:Epidermal growth factor-like domain |
| NDP | NDP, norrin cystine knot growth factor | GO:0045893~positive regulation of transcription |
| CHST11 | carbohydrate sulfotransferase 11 | GO:0002063~chondrocyte development |
| COMP | cartilage oligomeric matrix protein | IPR000742:Epidermal growth factor-like domain |
| FURIN | furin, paired basic amino acid cleaving enzyme | GO:0032902~nerve growth factor production |
| GPC1 | glypican 1 | GO:0040037~negative regulation of fibroblast growth factor receptor signaling pathway |
| HHIP | hedgehog interacting protein | GO:0040036~regulation of fibroblast growth factor receptor signaling pathway |
| MMP9 | matrix metallopeptidase 9 | GO:0045742~positive regulation of epidermal growth factor receptor signaling pathway |
| MFGE8 | milk fat globule-EGF factor 8 protein | IPR000742:Epidermal growth factor-like domain |
| PGF | placental growth factor | GO:0048010~vascular endothelial growth factor receptor signaling pathway |
| PDGFRB | platelet-derived growth factor receptor, beta polypeptide | GO:0038091~positive regulation of cell proliferation by VEGF-activated platelet derived growth factor receptor signaling pathway |
| RGCC | regulator of cell cycle | GO:0072537~fibroblast activation  GO:0090272~negative regulation of fibroblast growth factor production |
| SHISA2 | shisa family member 2 | GO:0040037~negative regulation of fibroblast growth factor receptor signaling pathway |
| THBD | Thrombomodulin | IPR000742:Epidermal growth factor-like domain |
| THBS3 | thrombospondin 3 | IPR000742:Epidermal growth factor-like domain |
| TGFBI | transforming growth factor, beta-induced, 68kDa |  |
| VCAN | Versican | IPR000742:Epidermal growth factor-like domain |

### 
