# Supplementary material for: Biomarkers for tissue engineering of the tendon-bone interface
Source: PLoS One. 2018 Jan 3;13(1):e0189668. doi: 10.1371/journal.pone.0189668 (PMC5751986; doi:10.1371/journal.pone.0189668)
Supplement: S3 Table — Candidates with transcription factor or growth factor activity were identified within the transcripts that were enriched in tendon compared to enthesis. (DOCX) [file pone.0189668.s003.docx]

**Biomarkers for tissue engineering of the tendon-bone interface**

Lara A. Kuntz^1,2,*^, Leone Rossetti^2^, Elena Kunold^3^, Andreas Schmitt^1^, Ruediger von Eisenhart-Rothe^1^, Andreas R. Bausch^2^, Rainer H. Burgkart^1,*^

^1^ Klinik für Orthopädie und Sportorthopädie, Klinikum rechts der Isar, Technische Universität München, D-81675 München, Germany

^2^ Lehrstuhl für Zellbiophysik, Technische Universität München, D-85748 Garching, Germany.

^3^ Center for Integrated Protein Science (CIPSM), Department of Chemistry, Technische Universität München, D-85747 Garching, Germany.

*to whom correspondence should be addressed: [kuntz@tum.de](mailto:kuntz@tum.de) and [burgkart@tum.de](mailto:burgkart@tum.de)

# Supplement

### S3 Table: Tendon transcription factors and growth factors

Candidates with transcription factor or growth factor activity were identified within the transcripts that were enriched in tendon compared to enthesis.

Table S3: Transcription factors and growth factors identified to be enriched in tendon compared to enthesis.

| **ID** | **Gene Name** | **GO molecular function terms (selected) or InterPro domain** |
| --- | --- | --- |
| **Transcription factors** |  |  |
| ENSSSCG00000016795 | brain abundant membrane attached signal protein 1 (BASP1) | GO:0003714~transcription corepressor activity |
| ENSSSCG00000028635 | general transcription factor II-I repeat domain-containing protein 2 (LOC100620992) | GO:0000981~RNA polymerase II transcription factor activity |
| ENSSSCG00000013511 | hepatoma-derived growth factor-related protein 2 (LOC100526035) | GO:0003677~DNA binding |
| ENSSSCG00000008624 | lipin 1 (LPIN1) | GO:0003713~transcription coactivator activity |
| ENSSSCG00000027115 | transcription initiation factor TFIID subunit 1-like (LOC100620926) | GO:0043565~sequence-specific DNA binding |
| CEBPA | CCAAT/enhancer binding protein alpha | GO:0000975~regulatory region DNA binding |
| CD3D | CD3d molecule | GO:0003712~transcription cofactor activity |
| ERCC3 | ERCC excision repair 3, TFIIH core complex helicase subunit | GO:0000166~nucleotide binding |
| EYA2 | EYA transcriptional coactivator and phosphatase 2 | GO:0004721~phosphoprotein phosphatase activity |
| ISL1 | ISL LIM homeobox 1 | GO:0000988~transcription factor activity, protein binding |
| KLF11 | Kruppel like factor 11 | GO:0000975~regulatory region DNA binding |
| MLXIPL | MLX interacting protein like | GO:0000976~transcription regulatory region sequence-specific DNA binding |
| SATB1 | SATB homeobox 1 | GO:0000976~transcription regulatory region sequence-specific DNA binding |
| YBX3 | Y-box binding protein 3 | GO:0000976~transcription regulatory region sequence-specific DNA binding |
| ZFP37 | ZFP37 zinc finger protein | GO:0001071~nucleic acid binding transcription factor activity |
| ATF3 | activating transcription factor 3 | GO:0000975~regulatory region DNA binding |
| ATOH8 | atonal bHLH transcription factor 8 | GO:0001071~nucleic acid binding transcription factor activity |
| BATF | basic leucine zipper ATF-like transcription factor | GO:0000975~regulatory region DNA binding |
| CRYM | crystallin mu | GO:0000988~transcription factor activity |
| CUX2 | cut like homeobox 2 | GO:0000975~regulatory region DNA binding |
| ESRRB | estrogen related receptor beta | GO:0001071~nucleic acid binding transcription factor activity |
| FOXP2 | forkhead box P2 | GO:0000975~regulatory region DNA binding |
| HIF3A | hypoxia inducible factor 3 alpha subunit | GO:0000981~RNA polymerase II transcription factor activity |
| IRF5 | interferon regulatory factor 5 | GO:0000975~regulatory region DNA binding |
| MED7 | mediator complex subunit 7 | GO:0000988~transcription factor activity |
| MYOC | myocilin | GO:0000988~transcription factor activity |
| NR2F1 | nuclear receptor subfamily 2 group F member 1 | GO:0000981~RNA polymerase II transcription factor activity |
| NR4A3 | nuclear receptor subfamily 4 group A member 3 | GO:0000975~regulatory region DNA binding, |
| PPARG | peroxisome proliferator activated receptor gamma | GO:0000989~transcription factor activity |
| PROX1 | prospero homeobox 1 | GO:0000975~regulatory region DNA binding |
| TSHZ2 | teashirt zinc finger homeobox 2 | GO:0000981 RNA polymerase II transcription factor activity |
| ZNF711 | zinc finger protein 711 | GO:0000976 transcription regulatory region sequence-specific DNA binding |
| **Growth factor activity** |  |  |
| ENSSSCG00000013511 | hepatoma-derived growth factor-related protein 2 (LOC100526035) | GO:0003677~DNA binding |
| ADAM19 | ADAM metallopeptidase domain 19 | IPR000742:Epidermal growth factor-like domain |
| ADAM22 | ADAM metallopeptidase domain 22 | IPR000742:Epidermal growth factor-like domain |
| EPHA3 | EPH receptor A3 | IPR009030:Insulin-like growth factor binding protein |
| EPHA5 | EPH receptor A5 | IPR009030:Insulin-like growth factor binding protein |
| FAT3 | FAT atypical cadherin 3 | IPR000742:Epidermal growth factor-like domain |
| KAZALD1 | Kazal type serine peptidase inhibitor domain 1 | IPR000867:Insulin-like growth factor-binding protein |
| RSPO2 | R-spondin 2 | IPR009030:Insulin-like growth factor binding protein |
| HMCN2 | hemicentin 2 | IPR000742:Epidermal growth factor-like domain |
| HGF | hepatocyte growth factor | IPR027284:Hepatocyte growth factor |
| IGFBP4 | insulin like growth factor binding protein 4 | GO:0005520~insulin-like growth factor binding |
| IGFALS | insulin like growth factor binding protein acid labile subunit | GO:0019838~growth factor binding |
| IGFBP5 | insulin-like growth factor binding protein 5 | GO:0019838~growth factor binding |
| LEP | leptin | GO:0008083~growth factor activity |
| MMRN1 | multimerin 1 | IPR000742:Epidermal growth factor-like domain |
| OIT3 | oncoprotein induced transcript 3 | IPR000742:Epidermal growth factor-like domain |
| PLAU | plasminogen activator, urokinase | IPR000742:Epidermal growth factor-like domain |
| PDGFRB | platelet-derived growth factor receptor, beta polypeptide | GO:0005017~platelet-derived growth factor-activated receptor activity |
| PTGS1 | prostaglandin-endoperoxide synthase 1 | IPR000742:Epidermal growth factor-like domain |
| PTGS2 | prostaglandin-endoperoxide synthase 2 | IPR000742:Epidermal growth factor-like domain |
| SCUBE2 | signal peptide, CUB domain and EGF like domain containing 2 | IPR000742:Epidermal growth factor-like domain |
| SLIT2 | slit guidance ligand 2 | IPR000742:Epidermal growth factor-like domain |
| SVEP1 | sushi, von Willebrand factor type A, EGF and pentraxin domain containing 1 | IPR000742:Epidermal growth factor-like domain |
| TENM1 | teneurin transmembrane protein 1 | IPR000742:Epidermal growth factor-like domain |
| TENM3 | teneurin transmembrane protein 3 | IPR000742:Epidermal growth factor-like domain |
| VEGFD | vascular endothelial growth factor D | GO:0008083~growth factor activity |
| VWCE | von Willebrand factor C and EGF domains | IPR000742:Epidermal growth factor-like domain |
