# Supplementary material for: Biomarkers for tissue engineering of the tendon-bone interface
Source: PLoS One. 2018 Jan 3;13(1):e0189668. doi: 10.1371/journal.pone.0189668 (PMC5751986; doi:10.1371/journal.pone.0189668)
Supplement: S4 Table — Candidates with transcription factor or growth factor activity were identified within the transcripts that were enriched in cartilage compared to enthesis. (DOCX) [file pone.0189668.s004.docx]

**Biomarkers for tissue engineering of the tendon-bone interface**

Lara A. Kuntz^1,2,*^, Leone Rossetti^2^, Elena Kunold^3^, Andreas Schmitt^1^, Ruediger von Eisenhart-Rothe^1^, Andreas R. Bausch^2^, Rainer H. Burgkart^1,*^

^1^ Klinik für Orthopädie und Sportorthopädie, Klinikum rechts der Isar, Technische Universität München, D-81675 München, Germany

^2^ Lehrstuhl für Zellbiophysik, Technische Universität München, D-85748 Garching, Germany.

^3^ Center for Integrated Protein Science (CIPSM), Department of Chemistry, Technische Universität München, D-85747 Garching, Germany.

*to whom correspondence should be addressed: [kuntz@tum.de](mailto:kuntz@tum.de) and [burgkart@tum.de](mailto:burgkart@tum.de)

# Supplement

### S4 Table: Cartilage transcription factors and growth factors.

Candidates with transcription factor or growth factor activity were identified within the transcripts that were enriched in cartilage compared to enthesis.

Table S4: Transcription factors and growth factors that are enriched in cartilage compared to enthesis.

| **ID** | **Gene Name** | **GO molecular function terms (selected) or InterPro domain** |
| --- | --- | --- |
| **Transcription factors** |  |  |
| ENSSSCG00000013049 | REST corepressor 2 (RCOR2) | GO:0001071~nucleic acid binding transcription factor activity |
| GLIS1 | GLIS family zinc finger 1 | GO:0003702~RNA polymerase II transcription factor activity |
| NKX3-2 | NK3 homeobox 2 | GO:0003700~transcription factor activit |
| NKX6-1 | NK6 homeobox 1 | GO:0003700~transcription factor activit |
| CYTL1 | cytokine-like 1 | GO:0016563~transcription activator activity |
| SATB1 | SATB homeobox 1 | GO:0003700~transcription factor activity |
| DLX3 | distal-less homeobox 3 | GO:0003700~transcription factor activity |
| EGR2 | early growth response 2 | GO:0003700~transcription factor activity |
| EGR3 | early growth response 3 | GO:0030528~transcription regulator activity |
| ETV5 | ets variant 5 | GO:0003700~transcription factor activity |
| FOXA3 | forkhead box A3 | GO:0003700~transcription factor activity |
| **Growth factors** |  |  |
| ENSSSCG00000030998 | WNT inhibitory factor 1 (WIF1) | IPR000742:Epidermal growth factor-like domain |
| ENSSSCG00000027367 | fibroblast growth factor receptor 2 (FGFR2) | GO:0005007~fibroblast growth factor-activated receptor activity |
| ENSSSCG00000001536 | signal peptide, CUB and EGF-like domain-containing protein 3 (LOC100152993) | IPR000742:Epidermal growth factor-like domain |
| ENSSSCG00000001695 | vascular endothelial growth factor A (VEGFA) | GO:0070851~growth factor receptor binding |
| WISP3 | WNT1 inducible signaling pathway protein 3 | GO:0008083~growth factor activity |
| BMP7 | bone morphogenetic protein 7 | GO:0008083~growth factor activity |
| FGF1 | fibroblast growth factor 1 (acidic) | GO:0008083~growth factor activity |
| FGFR4 | fibroblast growth factor receptor 4 | GO:0005007~fibroblast growth factor receptor activity |
| GDF5 | growth differentiation factor 5 | GO:0008083~growth factor activity |
| IGFBP5 | insulin-like growth factor binding protein 5 | GO:0019838~growth factor binding |
| LIF | leukemia inhibitory factor (cholinergic differentiation factor) | GO:0008083~growth factor activity, |
| MACC1 | metastasis associated in colon cancer 1 | GO:0008083~growth factor activity |
| TGFA | transforming growth factor, alpha | GO:0008083~growth factor activity |
